# Bioadhesion in the oral cavity and approaches for biofilm management by surface modifications

**DOI:** 10.1007/s00784-020-03646-1

**Published:** 2020-10-27

**Authors:** Torsten Sterzenbach, Ralf Helbig, Christian Hannig, Matthias Hannig

**Affiliations:** 1 Clinic of Operative and Pediatric Dentistry, Medical Faculty Carl Gustav Carus, Technische Universität Dresden, Fetscherstraße 74, 01307 Dresden, Germany; 2 Max Bergmann Center of Biomaterials, Leibniz-Institut für Polymerforschung Dresden e.V., Hohe Straße 6, 01069 Dresden, Germany; 3 Clinic of Operative Dentistry, Periodontology and Preventive Dentistry, University Hospital, Saarland University, Building 73, 66421 Homburg/Saar, Germany

**Keywords:** Oral biofilms, Biofilm management, Low-fouling surfaces, Nanostructured surfaces, Textured surfaces, Pellicle

## Abstract

**Background:**

All soft and solid surface structures in the oral cavity are covered by the acquired pellicle followed by bacterial colonization. This applies for natural structures as well as for restorative or prosthetic materials; the adherent bacterial biofilm is associated among others with the development of caries, periodontal diseases, peri-implantitis, or denture-associated stomatitis. Accordingly, there is a considerable demand for novel materials and coatings that limit and modulate bacterial attachment and/or propagation of microorganisms.

**Objectives and findings:**

The present paper depicts the current knowledge on the impact of different physicochemical surface characteristics on bioadsorption in the oral cavity. Furthermore, it was carved out which strategies were developed in dental research and general surface science to inhibit bacterial colonization and to delay biofilm formation by low-fouling or “easy-to-clean” surfaces. These include the modulation of physicochemical properties such as periodic topographies, roughness, surface free energy, or hardness. In recent years, a large emphasis was laid on micro- and nanostructured surfaces and on liquid repellent superhydrophic as well as superhydrophilic interfaces. Materials incorporating mobile or bound nanoparticles promoting bacteriostatic or bacteriotoxic properties were also used. Recently, chemically textured interfaces gained increasing interest and could represent promising solutions for innovative antibioadhesion interfaces. Due to the unique conditions in the oral cavity, mainly in vivo or in situ studies were considered in the review.

**Conclusion:**

Despite many promising approaches for modulation of biofilm formation in the oral cavity, the ubiquitous phenomenon of bioadsorption and adhesion pellicle formation in the challenging oral milieu masks surface properties and therewith hampers low-fouling strategies.

**Clinical relevance:**

Improved dental materials and surface coatings with easy-to-clean properties have the potential to improve oral health, but extensive and systematic research is required in this field to develop biocompatible and effective substances.

## The oral microbiota in health and disease

The oral bacterial community consists of more than 1000 different bacterial species and an estimated number of around 20 billion residents [1]. Most of them (≈ 96%) belong to the phyla of *Firmicutes*, *Actinobacteria*, *Proteobacteria*, *Fusobacteria*, *Bacteroidetes*, and *Spirochaetes* [2, 3]. Many of them are capable of colonizing both nonshedding surfaces of teeth (enamel or dentin) as well as epithelial mucosal surfaces. There they can form biofilms (plaque), which are highly variable in their composition depending on the specific surface, the particular location within the oral cavity, and the overall oral health status of the individual subjects, but also on environmental conditions like carbohydrate intake or flow of gingival crevicular fluid [4, 5]. Within healthy individuals, the host and microbial communities generally live in a homeostatic balance, and the oral microbiota serves many beneficial functions to the host. For example, it provides colonization resistance toward the settlement by pathogenic microorganisms [6]. Colonization resistance is mediated by several factors like competition for substrates, generating an inhibitory microenvironment for settlement of pathogens, release of antibactericidal substances, and stimulation of the host immune system (recently reviewed in [7]). However, many factors can disrupt this fragile equilibrium. This results in an imbalance (dysbiosis) of the microbiota ultimately leading to selection and enrichment of pathobionts [8]; for example, poor oral hygiene but also inflammatory and autoimmune diseases, immunodeficiency disorders, diet rich in low molecular carbohydrates, and many more health issues can disturb a healthy oral microbiota (recently reviewed in [9]). The breakdown of carbohydrates by *Streptococcus mutans* and other microbial pathogens can lead to an acidification of tooth surfaces. This may result in cavities due to demineralization and dissolving hard tissues of the teeth [10]. The onset of inflammation leads to the development of gingivitis [11]. This is caused by biofilm formation at the gingival margin. Colonization and inflammation of the subgingival pocket and the gingival crevice does not necessarily lead to the development of periodontitis, but rather to a change in environmental conditions, followed by an ecological shift toward Gram-negative and proteolytic bacteria that trigger immunological reactions that can lead to the clinical signs of periodontitis [12, 13]. Unlike gastrointestinal disorders, the onset of periodontitis is characterized by an increase in microbial diversity [14, 15]. However, most oral diseases are not caused by isolated infections with specific pathogens but are rather an intrinsic interplay between the host, keystone pathogens, and polymicrobial synergy and dysbiosis (PSD) [16, 17]. Nevertheless, a myriad of species have been connected to periodontal diseases, with the most frequent ones being *Fusobacterium nucleatum*, *Aggregatibacter actinomycetemcomitans*, *Prevotella intermedia*, *Porphyromonas gingivalis*, or *Tannerella forsythia* [18, 19].

However, the oral microbiota and related biofilm development do not only pose a major health issue on natural dental or soft tissue but also on artificial dental materials (e.g., resin-based composite fillings, crowns, dentures, or implants). This may lead to inflammation and destruction of soft and hard tissues surrounding dental implants. These conditions can develop into mucositis and peri-implantitis similar to gingivitis and periodontitis [20]. Also biofilm formation at the margins of dental restorations can lead to secondary caries [21]. Therefore, the development of artificial dental materials with reduced bacterial colonization or “easy-to-clean” properties is of outstanding importance for oral health. In this article, we will present and discuss the background as well as current and potential new strategies for the management of biofilm formation in the oral cavity based on surface modifications.

## Physicochemical basics of adhesion and adsorption

Attachment of proteins or microorganisms to surfaces is a multifaceted process and is mediated by a plethora of physical and chemical interactions. In the following chapter, we will present a brief overview of the processes involved in adhesion of both proteins and microorganisms to surfaces in general.

### Attachment of proteins to surfaces

**Fig. 1:**
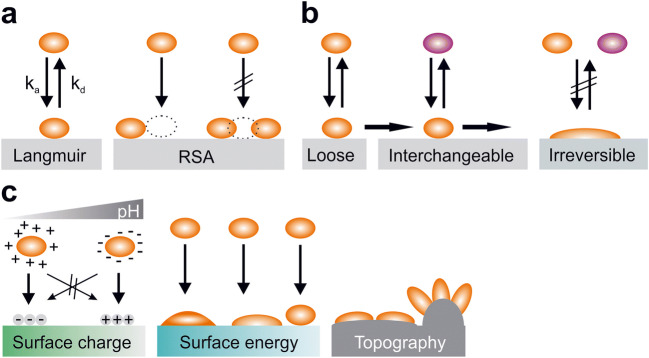
Interaction and adsorption of proteins to surfaces. **a** Illustration of the Langmuir and RSA (random sequential adsorption) models (*k*_a_ adsorption constant, *k*_d_ desorption constant). **b** Depending on the state and properties, proteins can loosely, interchangeably, or irreversibly attach to surfaces. **c** Factors that influence protein adsorption are, among others, properties of the protein(s), pH of the surrounding medium, and surface properties like surface charge, surface energy, or topography

In general, the Langmuir and the RSA (random sequential adsorption) model originally described basic protein adsorption to surfaces (Fig. 1a) [22]. Both models in its basic form cover adsorption of proteins in aqueous solutions to solid surfaces and assume that adsorption is reversible. In the Langmuir model, it is furthermore assumed that adsorbed molecules do not interact with each other, while the RSA model describes the probability of a new particle adsorbing to a surface in the presence of previously adsorbed particles. The Langmuir model then predicts the change of surface coverage over time as $\frac{\mathrm{d}\theta }{\mathrm{d}t}={k}_{\mathrm{a}}{C}_{\mathrm{b}}\left(1-\theta \right)-{k}_{\mathrm{d}}\theta$, where *θ* is the surface coverage, *k*_a_ and *k*_d_ are the adsorption and desorption constants, and *C*_b_ is the bulk concentration of the adsorbing molecule. The actual surface concentration *Ѓ* is then calculated as $\acute{\varGamma}={\acute{\varGamma}}_{\mathrm{max}}\theta ={\acute{\varGamma}}_{\mathrm{max}}\frac{K{C}_{\mathrm{b}}}{1+K{C}_{\mathrm{b}}}$, with *K* being the associated equilibrium constant [23]. In the RSA model, a function *Φ* (*θ*) is added which describes the probability function that new particles can adsorb in the presence of previously adsorbed particles [24]. This can then be described as $\frac{\mathrm{d}\theta }{\mathrm{d}t}={k}_{\mathrm{a}}{C}_{\mathrm{b}}\varPhi \left(\theta \right)-{k}_{\mathrm{d}}\theta$. In reality, the situation becomes more complex since proteins can either be loosely, interchangeably, or irreversibly bound to substrate surfaces (Fig. 1b). This is often accompanied by conformational changes of these proteins. Often, these conformational changes are accompanied by alterations in properties like, e.g., enzymatic or adhesive activities [25–28]. We will not go into details for these models in the context of this article, but they were recently nicely reviewed by Kim [22] and Sanfeld et al. [29].

In competitive adsorption situations with multicomponent solutions, it becomes more complex since various proteins will be present with different concentrations and different adsorption and desorption constants to surfaces. More abundant proteins will adsorb to surfaces first but can be replaced by less abundant ones with higher adsorption affinity. This can lead to sequential absorbance maximums of different proteins over time until an equilibrium is reached (Vroman effect) [30, 31]. Furthermore, in multicomponent solutions, protein–protein interactions will lead to the formation of homo- or heteromeric complexes [32].

External factors that modulate adherence to surfaces are especially the pH and ionic strength of the surrounding medium. Around the isoelectric point, electrostatic protein–protein repulsions are minimized. At pH values higher or lower than the isoelectric point, migration of proteins toward charged surfaces is maximized in the case of opposite charges and minimized in the case of similar charges between proteins and surfaces (Fig. 1c) [33]. High ionic strength also reduces electrostatic interactions between charged sites and increases the likelihood of protein aggregation because of surface charge neutralization [34].

Furthermore, the properties of the surface modulate adhesive behaviors of proteins (Fig. 1c). Factors that modify adsorption are among others surface free energy, charge, polarity, or morphology. In general, proteins tend to adhere stronger to surfaces with higher surface energy, to charged surfaces, and to nonpolar surfaces [35]. Although most proteins adhere better to slightly hydrophobic compared with hydrophilic surfaces, superhydrophobic surfaces generally yield reduced protein adsorption [36]. Depending on the properties of the protein and surface, adsorption of proteins is often also accompanied by orientational and conformational changes that can influence the properties of the protein [37, 38].

### Initial attachment of microorganisms to surfaces

The process of microbial adhesion can be divided into different steps: transport of the microorganisms to the surface, reversible adhesion to the surface, transition to irreversible adhesion, and emerging biofilm formation. Under flow chamber conditions, the initial transport of bacteria to surfaces is governed by the theoretical deposition rate according to the Smoluchowski–Levich (SL) approximation: ${j}_0^{\prime }=0.538\ \frac{D\infty C}{r}\left(\frac{h\mathrm{Pe}}{x}\right)1/3$, where *D*_*∞*_ is the bacterial diffusion coefficient, *C* is the bacterial concentration, Pe is the ratio between convection and diffusion, *r* is the hydrodynamic radius of the bacterium, and *x* is the distance from the inlet of the flow displacement system [39]. It should be noted that the contribution of gravity and interactions between depositing bacteria and the substrate surface is neglected in this equation. Real deposition rates are vastly influenced by factors like surface charge, motility, and surface coverage.

**Fig. 2:**
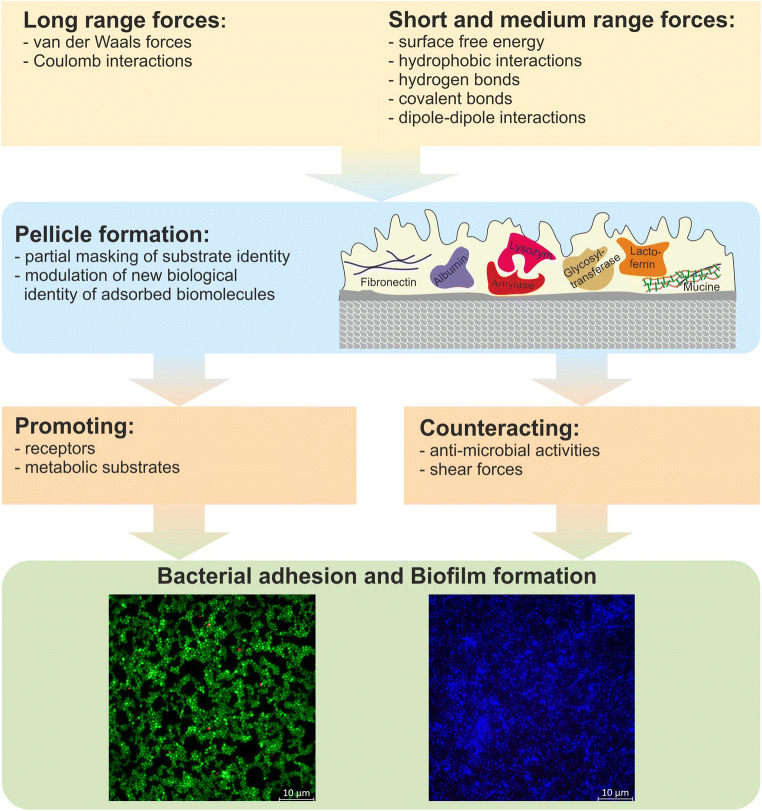
Interactions influencing bioadhesion and biofilm formation in the oral cavity. Different short, medium, and long range forces influence adhesion of bacteria to surfaces. The pellicle masks some of these properties while also providing new ones. Providing receptors and metabolic substrates promote, while shear forces and antimicrobial activities counteract bacterial adhesion and biofilm formation (modified according to Hannig et al. [41])

Nonspecific forces involved in reversible attachment of bacteria to surfaces can be grouped into short to medium (e.g., surface free energy, hydrophobic and dipole–dipole interactions, hydrogen, and covalent bonds) and long range forces (van der Waals forces and Coulomb interactions). Importantly, surfaces in the oral cavity are additionally covered by the acquired pellicle, which modulates surfaces and offers receptors, i.e., specific binding sites, but also counteracts adhesion by antimicrobial activities as discussed later (“The acquired oral pellicle” section). In addition, strong shear forces in the oral environment influence adhesion [40] (Fig. 2).

**Fig. 3:**
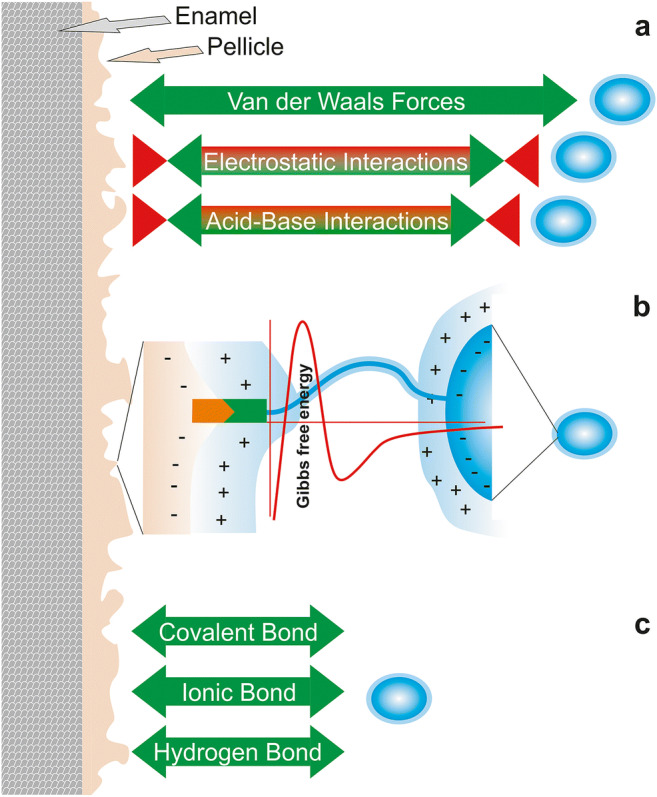
Initial bacterial adhesion to a pellicle-coated surface. **a** Initial bacterial approach and adhesion to surfaces is mediated by both attractive (green) and repulsive (red) short and long range forces [41]. **b** Depicted is the Stern layer surrounding the pellicle-coated surface and bacteria and the Gibbs free energy resulting from the sum of different forces (red curve) in relation to the distance between a bacterium and the surface. Small structures like fimbriae or flagella can overcome repulsive forces [43]. **c** The establishment of covalent, ionic, and hydrogen bonds establishes firm bonds between bacteria and the surface [44]

Classically, bacterial attachment to surfaces is explained by the DLVO (Derjaguin–Landau–Verwey–Overbeek) or the extended DLVO (XDLVO) theory (reviewed in [41, 42]). In short, in the extended DLVO theory, total interaction forces between bacteria and substrates (*F*_total_) are calculated as the sum of Lifshitz–van der Waals (*F*_LW_) forces, electrostatic interaction forces (*F*_EL_), and acid–base interaction (*F*_AB_) forces, which can be either attractive or repulsive (Fig. 3a). When plotting the sum of these forces to the distance between bacteria and surface, in very close proximity, forces will have a deep minimum and a secondary interaction minimum at a distance of 20–50 nm. But between these minima, forces will be strongly repellent leading to an energy barrier preventing attachment (Fig. 3b). Pure Brownian motion cannot generally pass this energy barrier so that bacteria remain trapped in the secondary minimum. In the original theory, bacteria were treated as colloidal round spheres, ignoring surface roughness of bacteria and surface appendages attached to the bacterial membrane (e.g., pili, fimbriae, flagella). It was suggested that surface appendages have a high probability to pierce through the energy barrier and thereby tether a bacterium to the surface [43, 45]. At this stage, a firm anchorage between the bacterium and the surface can be established involving covalent, ionic, or hydrogen bonding [44] (Fig. 3c). In this respect, it is important to note that adhesive strength is not mainly determined by contact area but rather by the amount and nature of contacts between surfaces and macromolecules on the bacterial surface [46, 47]. Recently, it was proposed that irreversible bacterial adhesion is the result of a multitude of reversible binding tethers that continuously detach and re-attach. Tight adhesion is achieved since these events do not happen simultaneously. Because these events do not occur at the same position, they lead to nanoscopic displacement of a bacterium thereby repositioning the bacteria on very small scales [48].

Bacteria that are in direct contact with a surface are subjected to cell wall deformation due to adhesion forces [49]. This leads to an increase in contact area and triggers differential gene expression in bacteria in close proximity to the surface [50]. These initial colonizers then signal to nearby bacteria by quorum sensing to trigger the switch from a planktonic to a biofilm stage in a larger population [51, 52]. Finally, also hydrodynamics and shear stress modulate adhesion of microorganisms to surfaces [53, 54].

### Influence of physicochemical properties on adhesion

**Fig. 4:**
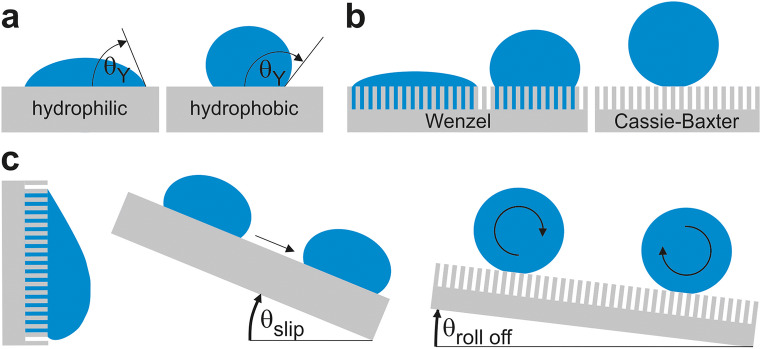
Interaction of water with different surfaces. **a** Water contact angles on different surfaces. Contact angles on hydrophilic surfaces are below 90°, while hydrophobic surfaces have contact angles of more than 90°. **b** Depiction of Wenzel and Cassie–Baxter wetting regimes. In the Wenzel state, cavities are fully wetted, while in the Cassie–Baxter state, air is trapped within cavities. **c** Water roll-off angles on hydrophilic and superhydrophobic surfaces. Hydrophilic surfaces possess relatively high roll-off angles, while superhydrophobic surfaces have roll-off angles of less than 5°

Bacteria tend to adhere best to slightly hydrophobic or hydrophilic surfaces, while strongly hydrophobic or hydrophilic surfaces generally lead to reduced adhesion (will be discussed later). Surface hydrophobicity or hydrophilicity correlates to surface wettability, which is often expressed by the contact angle (CA) of a water droplet on the substrate. Basically, the intrinsic CA at the boundary of the three phases (substrate, water, air) can be described by the Young’s equation $\cos \left({\theta}_Y\right)=\frac{\gamma \mathrm{SV}-\gamma \mathrm{SL}}{\gamma \mathrm{LV}}$ , where *γ*^SL^, *γ*^SV^, and *γ*^LV^ are the solid–liquid, solid–vapor, and liquid–vapor interfacial energies, respectively. The liquid–vapor interfacial energy is often referred to as surface tension of the liquid. Hydrophobic surfaces have an intrinsic CA greater than 90° and hydrophilic surfaces smaller than 90° (Fig. 4a).

In the intrinsic or actual CA, *θ*_*Y*_ is barely observable. One reason is that real surfaces are not completely flat. Therefore, the measured CA on real substrates can dramatically differ from *θ*_*Y*_, especially on very rough or textured surfaces. The Wenzel model [55] clarifies this behavior with a roughness value *r*, which is the ratio of actual and apparent surface area in a relation of the apparent (measured) CA (*θ*^∗^), and the (actual) intrinsic CA (*θ*_*Y*_), defined by the Young’s equation, cos(*θ*^∗^) = *r* cos(*θ*_*Y*_). Indeed, roughening of a surface can be considered as amplification of surface chemistry, i.e., it increases the CA of a water droplet on intrinsically hydrophobic surfaces and decreases it on intrinsically hydrophilic surfaces [56, 57].

So far, this scenario describes the wetting of chemically homogeneous substrates. Cassie and Baxter [58] extended this model for a droplet sitting on a multiphasic surface by cos(*θ*^∗^) = *f*_1_ cos(*θ*_*Y*1_) + *f*_2_ cos(*θ*_*Y*2_) + *f*_*i*_ cos(*θ*_*Yi*_), whereby *f*_*i*_ is the fraction of the different chemical phases, with ${\sum}_i{f}_i=1$, and *θ*_*Yi*_ being the respective intrinsic contact angles. One special case for very rough hydrophobic surfaces on which droplets can sit onto a so-called solid–air composite became very famous under the name superhydrophobicity. Here, the surface is only partially wetted (Cassie state)—in opposite to full wetting (Wenzel state)—and air is trapped in the surface cavities (Fig. 4b). This can be modeled by a droplet sitting on two phases, which can be described by cos(*θ*^∗^) = *f*_1_ cos(*θ*_*Y*_) + *f*_2_ cos(*θ*_Air_ = 180°) = *f*_1_ cos(*θ*_Y_) − *f*_2_ where *f*_1_ and *f*_2_ are the area fractions of solid and air under a drop on the substrate [59]. This wetting state is accompanied by very high CA (> 150°) and a highly repellent character, which can be defined by a critical tilt angle of the surface when the droplet starts to roll off (< 5°) (Fig. 4c). The low roll-off angle and the small topmost contact area on the rough surface reduce the temporal window and spatial possibilities for bioadhesion events for bacteria from a contaminated droplet. Furthermore, when immersed, the formation of the liquid–air interface between solid and liquid yields a protective layer, which is almost impossible to penetrate by bacteria and their appendages (fimbria, flagella). This prevents the settlement of microorganisms on the substrate and inhibits initial microbial adhesion.

## Bioadhesion in the oral cavity

### The acquired oral pellicle

**Fig. 5:**
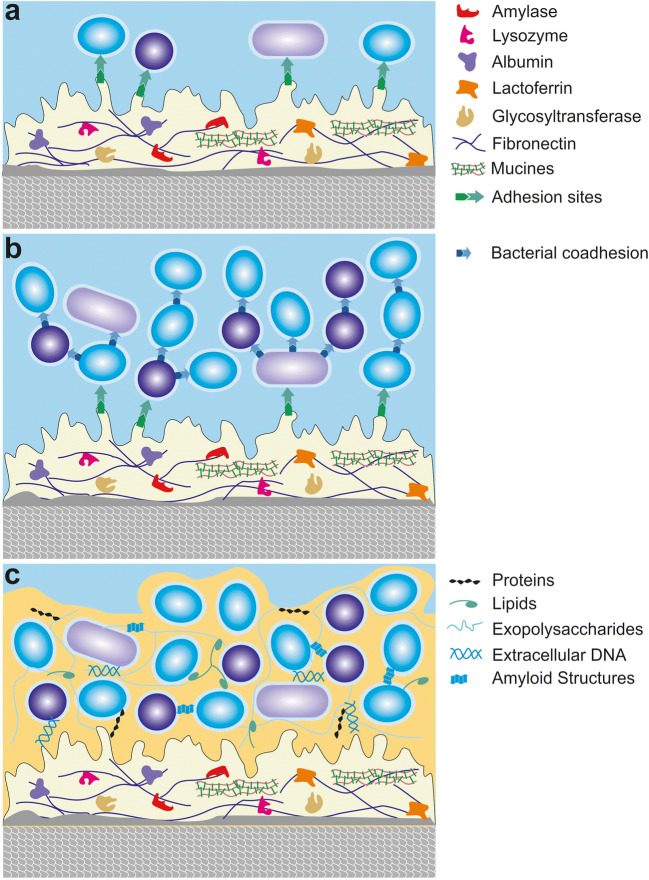
Overview of the acquired oral pellicle and biofilm formation in the oral cavity. **a** Depicted is the acquired oral pellicle on enamel composed of a thin basal pellicle and on top of it granular and globular structures. Early colonizers first adhere to lectins and other receptors on the acquired proteinaceous oral pellicle via specific adhesins in order to adhere tightly. **b** More microorganisms integrate into the developing biofilm structure by duplication or coadherence of further bacteria. **c** Depiction of a fully developed biofilm in the oral cavity. A multispecies biofilm is embedded into an extracellular matrix consisting among others of proteins, lipids, extracellular DNA, exopolysaccharides, and amyloid structures

Both soft (mucosal) and hard (enamel and dentine) tissues in the oral cavity are covered by the acquired oral pellicle within minutes (in more detail reviewed in [41, 60] (Fig. 5a). Almost instantaneously, an electron-dense pellicle layer is formed by adsorption of salivary proteins on the enamel surface [61, 62]. This is followed by a more continuous formation of a more complex globular layer [63]. The pellicle is mainly composed of selectively absorbed salivary proteins and peptides but also contains proteins and other macromolecules from gingival crevicular fluid, blood, bacteria, mucosa, and diet [62, 64–66]. Despite a large variability in individuals’ profiles, a core set of 68 proteins present in the pellicle proteome of 24 individuals was identified including among others antibacterial proteins (e.g., lysozyme, lactotransferrin, lactoperoxidase, and cystatins), lubricants (e.g., mucin 7), proteins promoting protein–substrate interactions (e.g., S100 family members, annexin A1, or elongation factor 2) or protein–protein interactions (e.g., 14-3-3 protein family or gamma-glutamyltransferase E), and proteins promoting pellicle integrity (e.g., carbonic anhydrase 6) [67, 68]. The pellicle provides many beneficial functions [61]. It serves as a lubricant and provides protection of the dental surface. It also serves as a protective layer against erosion by preventing decalcification of hard tissue [60]. Finally, the acquired pellicle contains several antibacterial components, among them relatively high concentrations of lysozyme and peroxidases. At the same time, the acquired pellicle also consists of many components that are beneficial to the adherence of bacteria and, henceforth, biofilm formation [69]. Especially ligands for bacterial adhesins like glycolipids, fibrinogen, or collagen play a major role in this process. They serve as anchor points for the initial adhesion of pioneer organisms like different *Streptococcus* spp. or *Actinomyces* spp. [70, 71]. This leads to early phases of biofilm formation and by the interconnection with other species to the establishment of mature plaque (see next chapter). However, it is important to note that according to recent studies, the ultrastructure, enzyme activities, and proteomic profile of the acquired pellicle do not show major differences between otherwise healthy caries-active and inactive subjects [67, 72, 73].

Furthermore, the oral pellicle affects the physicochemical properties of surfaces (e.g., surface roughness, hydrophobicity/hydrophilicity, wettability). For example, pellicle formation leads to significantly altered water contact angles on resin-based composites (concise, occlusin, heliomolar) compared with untreated samples [74]. In addition, pellicle formation can level out surface roughness [75–78].

This may lead to modification of bacterial adherence properties and degree of biofilm formation on natural and artificial surfaces in the oral cavity [79, 80].

### Oral biofilms

When forming biofilms on oral nonshedding surfaces, bacteria first have to adhere to the pellicle-coated substrate (Fig. 5a). Specific adhesins generally mediate initial adhesion to these surfaces [41, 69]. Most bacteria possess a plethora of different adhesins that are grouped among others into fimbrial adhesins, pili, autotransported adhesins, or surface proteins, which vary strongly in their assembly mechanisms, structure, and appearance [81–83]. For example, many pathogenic oral bacteria possess adhesins binding to major components of the oral pellicle like agglutinin, amylase, fibrinogen, fibronectin, or mucins [84–88]. Furthermore, many bacteria possess collagen-binding proteins [89, 90]. It is important to note that the composition of biofilms on natural surfaces within the oral cavity varies strongly depending on the localization (e.g., sub- and supragingival plaque vs. buccal or gingival mucosal biofilms) and between individuals [5].

*Streptococci* as well as *Neisseria*, *Rothia*, *Actinomyces*, or *Veillonella* are frequently found among others in the early stages of plaque formation, while after just 6 h, more than 90 different species belonging to 40 genera and 7 phyla were detected in the in vivo supragingival oral dental biofilm [91] (Fig. 5b). Later on, secondary colonizers like *Fusobacterium nucleatum*, *Treponema* sp., *Porphyromonas gingivalis*, or *Aggregatibacter actinomycetemcomitans* can be frequently found [92]. Furthermore, a complex extracellular matrix develops consisting of secreted proteins, lipids, exopolysaccharides, extracellular DNA, and amyloid structures [4, 93] (Fig. 5c). These microbial communities form an intrinsic network of co-existence and interspecies coaggregation [94]. Different species within the oral biofilm coregulate the expression of adherence factors as well as other virulence factors [4]. Furthermore, nutritional cross-feeding as well as co-ordinated metabolism of complex substrates takes place within biofilm communities (reviewed in [1]). In addition, bacteria in the oral environment also coregulate the expression of virulence factors (reviewed in [1]). For instance, biofilm formation and virulence gene expression of *S. mutans* is modulated by the presence of specific oral bacteria like *Streptococcus oralis* or *Lactobacillus casei* [95]. In addition, the surface can affect the transcriptional profile of bacteria [96]. Although the microbiota on mucosal sites strongly overlaps with the microbiota on nonshedding surfaces, several differences exist on the phylum, family, and genus levels. For example, buccal or keratinized mucosal surfaces show a high abundance of *Streptococcus* and *Gemella* spp., the tongue dorsum is characterized by *Veillonella* spp., and dental plaque has *Corynebacteriaceae* spp. as a biomarker [97, 98]. On the species level, for example, *Streptococcus mitis* has an exceptionally high abundance on keratinized gingiva, and for example, *F. nucleatum* or *Actinomyces naeslundii* are mainly found on the teeth, *Fusobacterium periodonticum* or *Actinomyces graevenitzii* on the tongue, and *Fusobacterium* sp. HMT248 or *Haemophilus haemolyticus* on keratinized gingiva [5, 99].

### Biofilms and pellicle formation on conventional dental materials

Similar to natural oral substrates, restorative or prosthetic materials and devices like implants, dental fillings, or crowns are almost instantaneously covered by an acquired pellicle. Here, the pellicle also modulates the physicochemical properties of the materials [74–78]. Thereafter, as on natural dental structures, biofilms will be formed, which often lead to secondary caries, mucositis, or peri-implantitis and even the ultimate failure of these medical devices. This was reviewed in 2009 in [41]. In the following chapter, we will present a short summary and update on biofilm and pellicle formation on conventional dental materials.

#### Biofilm formation on restorative materials

A variety of different materials is routinely used for restoration and sealing of cavities as for example resin-based composite materials, amalgam, or glass ionomer cements as well as inlays, crowns, or fixed partial dentures made from gold alloys, ceramics, or cobalt chromium (CoCr) alloys. However, the development of secondary caries due to biofilm formation on these materials and especially at the marginal gaps is the most common cause for their failure [100]. Amalgam-based materials have been mostly substituted in recent years by resin-based composite materials. However, besides the many advantages of resin-based composite materials, they more often lead to secondary caries compared with classic amalgam-based materials [101, 102]. Unlike on resin-based composites, biofilms on amalgam surfaces consist of mostly nonviable bacteria probably due to the slow release of mercury and silver [103]. Additionally, dental resin-based composites tend to be more susceptible to decay due to acid produced by cariogenic bacteria [104]. However, it was also suggested that resin-based composite materials and amalgam do not show differences in biofilm formation or microbiota composition in a microcosm biofilm model, in situ or on freshly extracted teeth with secondary caries [105–108]. Also, biofilms developed on bovine enamel and on resin-based composites seem to differ only slightly in situ [109]. Glass ionomer cements have the ability to release fluorides over time in an acidified environment thereby offering protection from caries-promoting biofilm development and decreased acid production at least under in vitro conditions [110–112]. Biofilm formation by a mixture of salivary microorganisms was also reduced on glass ionomer cement materials compared with resin-based composite materials or amalgam [113]. In addition, in vitro and in situ glass ionomer cements seem to offer protection against demineralization [106, 114]. Both in vitro and in situ biofilm formation on ceramics are mainly influenced by the composition and roughness of the used materials [115–117].

Polished gold alloys accumulated less bacteria compared with natural teeth in vivo although the composition of the biofilms was comparable [118]. In addition, biofilms accumulated on gold surfaces showed a low viability [119]. However, according to a 14-day in situ study, biofilm formation on gold, titanium, or zirconium abutment materials did not show major differences [120]. Also, biofilm formation over 48 h did not differ significantly between gold and ceramic bracket materials [121]. Studies on biofilm formation on CoCr alloys used in dentistry are inconclusive although at least under in vitro conditions biofilm formation seems to be in a similar range to titanium [122–124].

#### Biofilm formation on dentures

A large percentage of denture wearers are affected by denture-associated stomatitis, which is mainly caused by *Candida albicans* [125], albeit also differences in denture-associated biofilms on the genus and species level of other microorganisms can be detected between patients affected by denture-associated stomatitis and healthy subjects [126]. Dentures are mostly manufactured from polymethylmethacrylate (PMMA). However, at least in vitro alternative materials like Molloplast B or Ufi-Gel showed lower colonization by *C. albicans* [127]. In addition, excess of cement for fixation of crowns favor the growth of periodontal microbiota [128], thereby cements on a zinc oxide eugenol basis seemed better suited than methacrylate cement for crowns cemented on implants [129, 130].

#### Biofilm formation on implants

Dental implants are generally made of titanium or zirconium. Areas of these dental abutments accessible to the oral microbiota are globally covered within a short time period with extensive biofilms after the lack of oral hygiene [131, 132]. This can lead to inflammatory lesions that may develop into peri-implant mucositis or peri-implantitis [20, 133]. Altered microbial communities characterize these infections. However, these altered communities can be quite different from the communities found in caries or gingivitis. The overall microbial diversity in peri-implantitis is lower compared with healthy teeth and is dominated by Gram-negative bacteria [134]. Species that generally cluster with peri-implantitis are among others *P. gingivalis*, *P. intermedia*, *Treponema denticola*, *T. forsythia*, and *Fretibacterium fastidiosum* [135–137]. However, the microbiota of biofilms formed on dental implants does not seem to differ between subjects with or without former periodontitis [132].

Some studies suggest that abutments made of zirconium generally accumulate less biofilm compared with titanium in vivo [138, 139] although other studies did not find differences between these two materials [140]. The composition of biofilm communities varied depending on implant materials in short-term (24 h) splint experiments [141, 142]. Furthermore, in a 6-month in vivo study, differences in the microbial community between titanium and zirconia implants could be detected [143]. Also, biofilm formation on titanium or zirconium abutments varies depending on surface modifications, roughness, and surface free energy although results are also often inconclusive [144]. In addition, it was suggested that titanium ions released due to degradation of implants’ surfaces promote microbial dysbiosis as well as inflammatory processes around dental implants [145, 146].

#### Pellicle formation on conventional dental materials

To a large degree, biofilm formation on all these conventional dental materials is modulated by the formation of the acquired salivary pellicle formed on these materials as already outlined before. It was shown already around 20 years ago that the ultrastructural appearance of the in situ formed pellicle on enamel and various restorative materials like amalgam, ceramics, cements, resin-based composites, or titanium is very similar [65, 147]. In concordance with these findings, early plaque formation on these materials only showed minor, less pronounced variations. However, distinct differences were observed between buccally and lingually mounted test pieces [147]. Also at least under in vitro conditions, the composition of a pellicle formed on titanium or zirconium did not vary significantly in its protein content, while minor differences were found to pellicles formed on hydroxyl apatite [148]. Furthermore, amylase and lysozyme activity in the pellicle formed on different materials did not differ significantly in situ indicating similar biological activity of the pellicle on different dental materials [149, 150]. Nevertheless, in vitro adsorption kinetics of individual isolated salivary proteins varied between gold, titanium, and silica surfaces [151]. Also, different compositions of titanium modulated the composition of in vitro formed salivary pellicles [152]. In addition, roughening of titanium surfaces increased protein adsorption at least under in vitro conditions [77].

In summary, conventional dental materials still pose the problem of often extensive biofilm formation even under generally good oral conditions. This is caused by properties of the deployed materials that may promote bacterial attachment or the lack of anti-adhesive or antimicrobial properties. Furthermore, the acquired oral pellicle that forms on all these materials masks surface properties of these materials and, despite antibacterial properties, also provides anchor points for bacterial attachment. As a conclusion, there is a much-needed demand for novel improved materials in dentistry with increased low-fouling properties but that are otherwise fulfilling their role in the substitution of natural dental structures.

## Approaches for biofilm management in dental research

As outlined before, conventional prosthetic and restorative materials as well as implants in the oral environment often pose the problem of extensive biofilm formation [120, 127, 128, 132, 153]. This leads in many cases to the failure of the devices resulting in oral diseases like secondary caries, periodontitis, or peri-implantitis. Hence, many different kinds of strategies were developed to decrease adherence and/or biofilm formation on artificial dental surfaces. These include materials that modulate adherence of microorganisms as well as “easy-to-clean” surfaces that prevent tight attachment and allow easy removal of adhered microorganisms [154, 155]. Furthermore, concepts were developed that allow killing (bactericidal) or growth inhibition (bacteriostatic) of microorganisms upon surface contact. Strategies comprise modulation of surface architecture and topography as well as chemical or mechanical modification of surface structures. Chemical modulations were applied to change surface energy (hydrophobic vs. hydrophilic), or charge and mechanical properties were controlled by alternation of the intrinsic material elasticity. Often, different strategies are combined, such as mechanically or chemically modulated topographies or incorporation of functionalized nanoparticles into basic matrices. Many of these strategies offer the advantage that they can be implemented well in dental laboratories. Also, removable dentures can also be modified at later time points by coating of the surfaces.

These approaches will be discussed in more detail in the following chapters. The review is designed as a narrative update to a previous review from 2009 [41]. It focuses mainly on novel strategies that have been tested under in vivo or in situ settings and with proven or potential clinical relevance in dental practice. We to most part excluded studies based on in vitro experiments that were only tested under in vitro settings or with unlikely clinical relevance.

### Biocompatibility of novel materials in dentistry

When designing novel strategies for the management of microbial adherence and colonization in the oral environment, it is important to keep in mind the biocompatibility of these materials. Biocompatibility refers to the question how these materials interact with the host. In general, it is assessed regarding biodegradation of materials, cytotoxicity toward eukaryotic and microbial cells, interaction with natural materials (e.g., teeth, bones) as well as inflammatory potentials, immunotoxicity, or mutagenicity [156, 157]. Especially in the oral environment, devices should often last for decades with high durability and low level of biodegradation. Therefore, assessment of biocompatibility should ideally also consider long-term effects, which is often difficult to evaluate.

For example, dental casting alloys are generally composed of often more than six different metals. Due to corrosion, these components can be released and interact with the host [158]. Many of these alloys and its metals, particularly heavy metals, show cytotoxicity toward cell lines and yeast [159–161]. Especially biodegradation of amalgam reached a lot of attention due to the biotoxicity of mercury [162]. Also, the remaining unpolymerized fractions from resin-based composite materials or light curing glass ionomer cements (e.g., methacrylates) can lead to cytotoxicity, immunotoxicity, or hypersensitivity [163, 164]. In addition, as outlined before, titanium ions released due to degradation of implants’ surfaces may promote microbial dysbiosis as well as inflammatory processes around dental implants [145, 146].

Similar problems may arise from the usage of nanoparticles. Functionalization of prosthetic and restorative materials can have beneficial effects on managing microbial colonization but as a downside exhibit unwanted side effects (recently reviewed in [165]). For example nanohydroxyapatite or TiO_2_ nanoparticles can lead to inflammatory responses and oxidative stress [166].

Nanostructured bactericidal surfaces often pose the problem that they are also detrimental to eukaryotic cells. Surface structures can pierce eukaryotic cells, which may lead to cell death or cell stress. Human fibroblast foreskin cells exhibited altered morphologies on 3D nanoposts [167]. However, other studies showed no effects of nanopores on attachment, morphology, and metabolic activity of fibroblasts [168].

### Surface roughness and topography

**Fig. 6:**
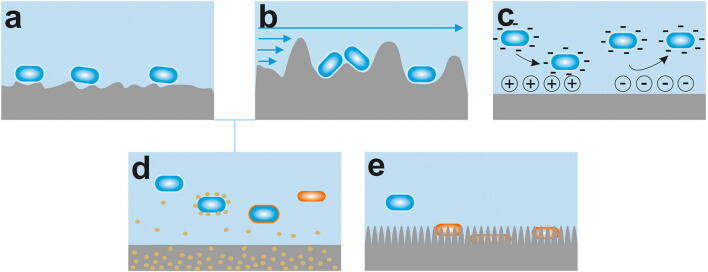
Interaction of bacteria with different surface properties. Rough surfaces increase the surface area (**a**) or offer protection against shear forces (**b**). **c** Since bacterial membranes are generally negatively charged, positively charged surfaces are generally attractant and negatively charged surfaces repellent. **d** Functionalized nanoparticles embedded into surfaces with anti-adhesive or antimicrobial properties can prevent attachment or proliferation of bacteria after surface contact or they are released into the surrounding environment. **e** Nanotextured superhydrophic surfaces are often bactericidal due to stretching of the membrane

Traditionally, surface roughness of dental restorative materials was reduced to limit bacterial adhesion and biofilm formation (recently reviewed in [169]). It is commonly believed that higher surface roughness influences bacterial attachment mainly by increasing the surface area (Fig. 6a). Additionally, it was suggested that higher surface roughness leads to higher microbial colonization since it offers shelter against shear forces especially at initial attachment (Fig. 6b) and because they are more difficult to clean compared with smooth surfaces [44, 76, 170]. Indeed, many in situ or in vivo studies showed that reduction of surface roughness reduced bacterial adhesion on commonly used implant materials. For example, reducing surface roughness of titanium or zirconia alloys reduced colonization and biofilm formation [171–174]. However, other in vivo or in situ studies contradict this opinion and suggest that modification of surface roughness only plays a modest role in altering bacterial adherence and biofilm formation [175–179]. Some in vivo and in vitro studies also indicate that surface roughness on dental materials only has a significant impact if the average height (*R*_a_) is larger than 0.2 μm [44, 180–182].

In this respect, it is important to remember that all surfaces exposed to the oral cavity are covered by the acquired pellicle within short amounts of time. As outlined before, the pellicle can level out surface roughness [75–78]. This might explain contradictory results between different studies depending on the exactly utilized model as well as between in vitro and in vivo studies [179]. In addition roughness is commonly defined by different parameters like average height (*R*_a_), root mean square roughness (*R*_q_), and ten-point height (*R*_z_) [183]. Depending on the applied parameters and methods for assessing roughness as well as the measured area, similar surfaces can end up having different “roughness values,” while surfaces with different topographies may have similar “roughness values” [184–186]. In addition, bacteria can modulate the expression of virulence genes depending on the surface topography. For example, *Escherichia coli* differentially expresses among others type 1 fimbriae and the Cpx two-component system, a general stress response system, when being grown on nanostructured gold surfaces at least under in vitro conditions [187, 188].

### Surface charge and energy

Similarly, charge and surface energy influence adherence of microorganisms. Since the outer layer of bacteria is negatively charged, they tend to adhere better to positively than negatively charged surfaces according to in vitro studies [189, 190] (Fig. 6c). Furthermore, bacteria tend to adhere best to surfaces with moderate wettability compared with highly hydrophobic or hydrophilic surfaces. In vitro studies suggest that in general water contact angles in the range of 40° to 130° seem to lead to the highest bacterial adhesion [191–193]. Accordingly, coating of stainless steel or alumina ceramic with hydrophobic hexadecyltrimethoxysilane or perfluorodecyltriethoxysilane reduced biofilm retention in a microcosm model using human saliva [194]. Similarly, coating of titanium or stainless steel surfaces with hydrophobic polytetrafluoroethylene strongly reduced biofilm formation in in situ approaches [195, 196]. In addition, coating of enamel or titanium with a low surface free energy nanocomposite (NANOMER) could successfully and strongly reduce biofilm formation in situ [155]. Coating with the nanocomposite reduced the thickness as well as the protein–protein adsorption and interaction forces of the acquired oral pellicle. This lead to easier removal of the pellicle and, henceforth, easier detachment of the overlying biofilm thereby promoting “easy-to-clean” properties.

Especially superhydrophobic surfaces tend to reduce bacterial adherence and biofilm formation. These often have microscaled structures on their surface that result in high contact angles. By this means, a superhydrophobic state is reached leading to a self-cleaning effect by rolling off water [197, 198]. This reduces bacterial colonization by simply washing off colonizing bacteria and developing biofilm structures. Furthermore, air becomes entrapped between nanoscale structures. Consequently, the contact area as well as the adhesive forces between bacteria and substrate is reduced. In recent years, many artificial superhydrophobic surfaces on, for example, aluminum, titanium, or polypyrene were developed that successfully reduced bacterial adhesion at least in vitro [199–201]. Although almost no data are available upon the suitability of these approaches for the oral cavity, these approaches justify further research for their applicability due to the nontoxic nature of this approach. However, it should be noted that some studies suggest that the effect of superhydrophobic structures may be short lived. The entrapped air-bubble layer can get lost over time since liquids penetrate the nanostructures (in more detail later). This may then actually promote bacterial adhesion due to increased surface areas [202, 203].

### Functionalization of dental materials with antimicrobial activities

Another strategy to reduce bacterial attachment and biofilm formation on dental implants as well as crowns or fillings is either chemical modification of the materials or the linkage with molecules with antimicrobial activity. A drawback of these approaches might be sustainability since chemical modifications or antimicrobial activities may get lost over time. We will give here an exemplary overview of studies with promising potential.

In several approaches, copper, silver, gold, iron oxide, or zinc oxide nanoparticles were incorporated into resin-based composites like chitosan or other matrices and showed antimicrobial activity at least in vitro [204–209]. They generally act by disruption of the bacterial membrane through physical interaction with the nanoparticles, generation of oxidative stress, and enhanced release of free metal ions (Fig. 6d) (reviewed in [210]). Incorporation of silver-doped bioactive glass into resin-based composites allowed remineralization of dental surfaces and protection against in vitro formed biofilms by *S. mutans* and *L. casei* [211]. Titan substrates modified with a thin layer of graphene oxide and silver nanoparticles also reduced adhesion with increased bactericidal activity toward *S. mutans*. Furthermore, expression of the genes encoding for the main subunit of type 1 fimbria FimA and for the glucosyltransferases GtfB and GtfC was significantly reduced [212]. Unfortunately, all these studies were conducted in vitro, while to our knowledge, no in vivo or in situ data are available yet. Also, modifications of PMMA were developed to reduce adherence of *Candida* spp. like incorporation of nanodiamonds or silver/bromide/cationic polymer nanocomposite (AgBr/NPVP) as well as coating with polyacrylic acid or polycationic acid [213–215].

Quaternary ammonium methylacrylates like dimethylaminododecyl methacrylate incorporated into glass ionomer cement could reduce biofilm formation and microbial viability in situ [216]. It also sustained its properties and showed minimal signs of aging even after incubation in water for at least 6 months, suggesting long-term stability in the oral environment [217]. Incorporation of chlorhexidine salts into glass ionomer cements could also significantly limit microbial counts under in vivo conditions in shorter-term studies up to 7 days, and even after 1 year, still a reduction could be observed [218, 219]. Similarly, resin-based composite materials containing octenidine dihydrochloride (ODH) strongly reduced biofilm formation as well as the fraction of viable microbial cells in situ [220]. Chitosan itself also has antimicrobial activities at acidic pH [221]. It could reduce biofilm formation in in vitro studies when being incorporated into resin-based composite materials [222]. Some studies also tried to successfully disrupt biofilm formation by impairing adhesion between *Streptococci* and *Porphyromonas* [223, 224]. Xu et al. [225] tested a combination of both chemical and topological modifications of surfaces using polyurethane with a pillared topography and a S-nitroso-N-acetylpenicillamine (SNAP) layer releasing NO. Although both treatments alone were able to reduce biofilm formation by *Staphylococcus epidermidis*, in combination, they showed a synergistic effect.

In summary, chemical modifications of surfaces with antimicrobial properties has promising potential. However, as already mentioned, sustainability of these structures or materials for prolonged time periods in the oral cavity has to be evaluated. Furthermore, biocompatibility of these approaches needs further attention especially in regard to long-term toxicity. In addition, long-term exposure to quaternary ammonium compounds and other antimicrobial substances may lead to the development of resistances in bacteria [226]. This may lead to selection for resistant bacteria in the oral cavity hence counteracting antifouling properties of the surfaces. If resistance genes are encoded on mobile elements, they may also spread to other species in the oral cavity [227]. Nevertheless, promising potential definitely warrants further research in this direction.

## A broader view on antibioadhesion strategies in life sciences

The former chapter gave an overview about the state of the art of biofilm management in dentistry, and it has been shown that there is still a need for improvements. Therefore, in the following chapter, we will elucidate antibioadhesion strategies in a broader context in order to figure out promising and appropriate tools for future application in the oral cavity. We will concentrate on research and perspectives of physicochemical interfacial properties considering the challenge of biocompatibility.

### Surface structure

Parameters like surface structuring and roughness were investigated for decades and a vast amount of publications exists, which describe their impact on bacterial colonization. Structural parameters were evaluated from the nanoscale (approximate size of molecules and cell appendages) up to the microscale (approximate size of cells or larger) [228, 229], thereby microscaled structures are mostly periodic topographies described by their structural features and nanoscaled structures usually are semiperiodic or random topographies described by roughness parameters [230, 231]. Structures much larger than cells do not directly have relevance for prevention of biofouling processes as they provide niches that can support undisturbed attachment and resistance against removal. But large structures can have a secondary advantage if they are part of a hierarchical structure in which they provide mechanical support against abrasion of smaller structural levels [232–234]. However, on simple surface structures, strong interactions between bacteria and substrate as well as clear adhesion patterns in the initial colonization phase could be observed if the structural dimensions (size and spacing) were in the cell size range [228, 230, 231, 235–240]. In contrast, for structure sizes slightly below the bacterial cell size (submicron scale), inhibition of the initial microbial adhesion could be observed [229, 241, 242]. For much smaller nanostructures, the reported effects differ strongly between being high and low adhesive [243, 244]. It has been shown that even the smallest differences in surface nanoroughness can strongly affect microbial cells’ attachment [245–250]. These variations must be connected with certain spacings of bacterial adhesion sites on the membrane or cell appendages, but there is no direct evidence in the literature so far. Additionally, nanostructured surfaces can alter protein adsorption in comparison to the flat material, because small curvature radii of structural features can influence protein conformation and disturb molecular alignment (Fig. 1c) [251]. Thus, depending on the native protein shape (such as globular for BSA vs. elongated for fibrinogen), small surface structures can either decrease or increase adsorption [252]. However, there are many contradictory experimental observations on the effect of micro- and nanostructured surfaces on biomolecular adsorption and bacterial colonization [228, 235, 236, 238–240, 253, 254]. These contradictions arise often due to inconsistent comparison of physicochemical properties of the samples and the use of differing biomolecules/bacterial strains as well as general differences between experimental setups/parameters. This illustrates clearly that systematic research on the effect of different scales on surface structures on bacterial colonization in an easily accessible clinically relevant environment (oral cavity) is strongly required.

### Surface free energy and elasticity

Controlling the surface chemistry (i.e., surface free energy and/or elasticity) of a substrate is another promising approach to control biofouling. Substrates with low and high surface free energies in particular have been shown to affect biomolecule adsorption and bacterial adhesion [255]. Hydrophilic surfaces featuring high surface free energies, such as poly(ethylene glycol) (PEG)-based coatings, can inhibit biofouling by steric repulsion due to chain compression and the “barrier” created by structured water associated with the PEG chains [256]. An opposite approach is the use of hydrophobic coatings with low surface free energies, sometimes called theta surfaces [257]. These surfaces do not inhibit adsorption/adhesion per se, but allow for an easy removal of adsorbates and biofilms [257]. Furthermore, the use of soft surfaces could reduce attachment of certain bacterial strains [258, 259]. However, there are also bacterial strains which adhere well to soft substrates, especially if specific adhesion sites for protein–protein or protein–saccharide interactions exist, i.e., on eukaryotic tissue, mucosa, or adsorbed protein layers on artificial substrates [260–263]. Nevertheless, there are bacterial strains which do not show significant changes of adhesion over different magnitudes of material stiffness [264].

### Bio-inspired strategies

In recent years, research on improved biomedical surfaces started to focus on mimicking natural (e.g., shark skin, lotus and taro leaves, cicadia wings, springtail’s cuticle, or pitcher plants) antifouling (reduced microbial adherence or attachment) or bactericidal (active killing of bacteria) surfaces (recently reviewed in [265]). The antifouling properties of these surfaces are generally explained by their hydrophobic nature combined with a nanopillared geometry leading to a superhydrophobic (“nonwetted”) state as explained by the Wenzel and Cassie–Baxter model and the so-called lotus effect [266–268]. In this state, only a very small contact exists between the contaminated liquid and the substrate, which reduces the spatial opportunity for bioadhesion, and liquids roll off immediately at the slightest tilt, which reduces the temporal opportunity for fouling processes. Repellency against aqueous media can be observed on many plants but also on arthropod cuticles. Springtails were found to be even more repellent as they can resist wetting against liquids with low surface tensions (oil, ethanol, etc.) [269, 270] by submicroscaled structures with overhanging cross-sectional profiles [271]. Another strategy for preventing bacterial colonization is the utilization of “slippery” covering layers on harder substrates found on pitcher plants [272, 273]. The slippery substrate strongly prevents bacterial adhesion, because there is almost no appropriate counterpart for adhesion in the slime.

Depending on the size and distribution of nanotopographies, surface structures can also act bactericidal than just prevent adhesion. The mechanism of cicadia wings and similar structures is explained by their small feature size (~ 50–100 nm) and the high aspect ratio [274–276]. This leads to stretching and puncture of the bacterial membrane and subsequent cell lysis either by suspension of the membrane between pillars (biophysical model) or by “tearing” of the membrane due to an increase in the surface contact area (thermodynamic model) [154, 277, 278] (Fig. 6e). While most of these structures were never applied to the oral cavity, exposure of springtail cuticles to saliva was tested in situ and a modulated process of initial bioadhesion was observed. In contrast to a pellicle homogenously coating all surface structures, there was at least in part the formation of proteinaceous membrane-like structures bridging the springtail’s cuticle nanostructures. As a result, springtails could modulate bacterial adhesion on short-term time points but lost its specific properties after exposure for a few hours [241]. This clearly indicates the relevance of early in situ experiments.

To transfer innovative biomimetic strategies to medical and dental surface devices, silicon- or titania-based nanostructured surfaces are increasingly evaluated for their suitability. For example, nanostructures were generated on silicon surfaces by ion etching via a silicon wafer ([279] or fabrication of nanocone-shaped diamonds on silicon substrates [280]. In vitro, these structures were able to kill *E. coli*, *Staphylococcus aureus*, or *Pseudomonas aeruginosa*. Titanium surfaces containing nanowires with a diameter of ca. 100 nm were efficient in killing motile bacteria like *P. aeruginosa*, *E. coli*, or *Bacillus subtilis* [275]. In similar approaches, nanoscale structures were introduced onto different materials like alumina surfaces thereby limiting bacterial attachment [281–283]. Nanopores of 20–25 nm were introduced onto stainless steel by electrochemical etching [168]. This modification leads to substantially reduced adhesion of *S. aureus* and *E. coli* while not affecting attachment, morphology, and metabolic activity of fibroblasts, suggesting good biocompatibility of these materials.

Unfortunately, most of these data were obtained under in vitro laboratory conditions, while these structures were rarely evaluated for their suitability in the oral cavity. However, micropatterning of titanium surfaces by laser etching leads to reduced biofilm formation in situ compared with machined or grit-blasted surfaces [284]. Also, few studies were conducted in the oral environment. However, Miao et al. showed reduced initial adhesion of the oral pathogen *S. mutans* to nanotextured compared with smooth or microtextured titanium surfaces while at the same time improving attachment of human gingival epithelial cells or fibroblasts [285]. Also, a nanotextured titanium surface with an average roughness of approximately 10 nm could reduce surface colonization of *A. actinomycetemcomitans* compared with untreated titanium surfaces [286].

Hence, promising in vitro results and a likely good biocompatibility of these materials definitely warrant further in vivo or in situ studies to elucidate their applicability in the oral cavity. Nanostructured implant materials mimicking natural antifouling materials definitely have the potential to efficiently modulate and reduce biofilm formation on oral medical devices and materials.

### Heterogeneous surface properties

Recently, materials, which combine different or even opposite physicochemical properties within a molecule, along a polymer chain or across a surface area came into the focus of intense research. Low bioadhesion could be shown for coatings with zwitterionic polymers like phosphorylcholin or polybetains [287–291]. The close proximity of negative and positive charges leads to a strong hydration which reduces protein adsorption. Furthermore, amphiphilic surfaces with nanometer-sized hydrophilic and hydrophobic domains were among others achieved by nanocomposites, block copolymers, hyperbranched fluorpolymer–PEG, and fluorpolymer–PDMS–PEG networks, whereas micrometer-sized domains were realized using a Janus-particle–based system [255, 292, 293]. Low cell adhesion and good fouling release properties of these bi- or multiphasic interfaces were reported to often outperform surfaces featuring just monophasic properties. For some of the tested surfaces, also reduced protein adsorption was reported. On chemically nanoscaled heterogeneous silica–zirconia surfaces, the net amount of adsorbed albumin was higher than predicted by the average of adsorption on both monophases [294]. Other research groups have reported an opposite effect on biphasic microscaled substrates [295, 296], but no convincing explanation of the altered adsorption had been presented. Polyelectrolyte multilayer coatings were found to reduce protein adsorption, whereby it was hypothesized that the formed nanoscaled domains and the low surface net charge are the origin of this effect [297]. However, it is plausible that spatial alterations of different chemical phases in the range of the protein sizes influence adsorption processes and conformational changes, analogous to surface structures with curvature radii in the nanoscale [297–300].

## Discussion

In recent years, a plethora of studies tested multitudes of approaches to construct novel low-fouling or antibactericidal surfaces to reduce attachment of microorganisms or biofilm formation on biomedical surfaces in dentistry and beyond. In an ideal situation, prosthetic dental materials should closely mimic natural dental structures without their downsides. They should be resistant to erosion by substances produced by the host and microbiota in the oral environment. In addition, most prosthetic materials in the oral cavity should function for years or even decades. Hence, low-fouling or antibactericidal activities must last for extended periods of time without deterioration or loss in functionality. This should be kept in mind especially for approaches employing chemical modifications or nanocarriers. While they may well work on short-term perspectives, their long-term viability is often questionable. Another important aspect is biocompatibility of restorative materials in the oral environment. It is important that they do not cause toxic reactions to the host both on a short- and long-term time span. Especially approaches employing chemical modifications, nanoparticles, or nanocarriers may present problems with biocompatibility. While some studies examined biocompatibility, many studies lack data in that direction. Importantly, novel materials must be moldable to be implemented into the existing dental environment without cracks and crevices. Other aspects to be considered are cost-effectiveness and acceptance by patients.

In the oral cavity, surfaces are almost instantly covered by the acquired pellicle [61, 147]. As outlined before, the presence of the pellicle may mask or alter structural properties of surfaces like roughness, wettability, and hydrophilicity or hydrophobicity [74–76, 78, 80]. Furthermore, the pellicle itself presents attachment points for the adhesion of microorganisms as well as possessing antimicrobial properties [61, 69]. Hence, surface modifications that work well in reducing biofilm formation in implants outside of the oral cavity (e.g., joint replacements) may not be efficient in the oral cavity due to the presence of the acquired oral pellicle. On the other hand, these phenomena make the oral cavity a perfect model to evaluate new strategies in an easily accessible and clinically relevant model.

In addition, studies employing similar approaches have often inconclusive or even contradictory results. Similar approaches may have a protective effect in some studies, while other studies showed no effect or even lead to enhanced microbial adhesion or biofilm formation (e.g., surface roughness) [171–179]. Reasons may often be the design of the study (e.g., in vitro vs. in situ or in vivo; models used for the study; or time frames). Structural alterations of surface properties also often affect several physical and chemical properties like roughness, surface wettability, hydrophobicity, or hydrophilicity [301]. This makes it often challenging to pinpoint specific effects on adhesion or biofilm formation to one specific property. Finally, different biophysical and biochemical properties (e.g., surface charge, Gram-positive or negative, arsenal of adhesins) of different microorganisms can lead to a change in biofilm community on different restorative materials [69, 189]. It is therefore important to consider that not only the degree of biofilm formation must be considered but also the composition of the community (pathogenic vs. commensal microorganisms).

Traditionally, many studies focused on altering surface properties of structures like surface roughness or surface charge or energy with varying success [155, 171, 173–175, 177–179, 194, 196]. In addition, incorporation of antimicrobially active metal ions is a common strategy to limit microbial settlement especially on resin-based composite materials [211, 212]. Nevertheless, many novel promising approaches appeared within the last years. Especially superhydrophobic nanotextured surfaces with both antifouling and/or antimicrobial activity inspired by nature appear promising [241, 284, 286]. In addition, many studies focused on incorporating nanoparticles with antimicrobial or anti-adhesive properties mainly into resin-based composite materials [216–220, 302]. The majority of these studies were conducted in vitro showing the general applicability of these approaches. However, more studies directly in the oral environment are necessary to prove “real-life” applicability in the mouth. In addition, long-term stability and biocompatibility were rarely evaluated. Still, these approaches are promising pathways for biofilm management on oral implants or resin-based composite materials and warrant further attention.

The majority of strategies for biofilm management focused on single strategies like easy-to-clean surfaces, bacteriostatic or bactericidal surface properties, or incorporation of antimicrobial substances via surface coupling or nanoparticles or carriers. While some approaches already combined different strategies [225], further improvement may well be achieved by increased combinatory approaches.

**Fig. 7:**
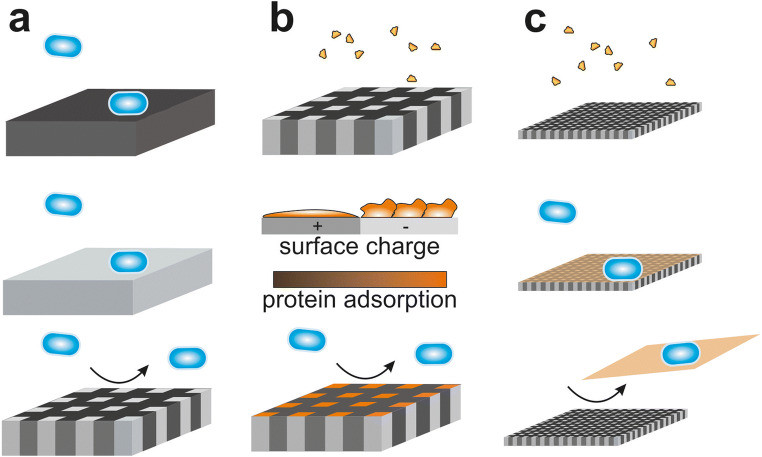
Interaction of bacteria and proteins with biphasic interfaces. **a** Bacteria can adhere to surfaces with various interfacial properties but might have problems to adapt to surfaces with alternating interfacial properties. **b** Protein adsorption transforms the synthetic textured identity into a biological alternating identity. **c** Nanotextures allow bioadhesion but might lead to easy removal

Overall, the main question arises which approaches warrant further research in respect to functionality, sustainability, and biocompatibility. While functionalization of surfaces with antimicrobial or anti-adhesive properties might be beneficial in a short-term perspective, sustainability and biocompatibility remain questionable. Modifying roughness or surface charges (wettability) also offered inconclusive results in a plethora of studies. While generation of nanostructured topographies to generate superhydrophobic and/or bactericidal surfaces showed promising results, the question about the long-term prospective of these approaches still remains. First, with few exceptions, these strategies were rarely evaluated in the oral cavity and beneficial protective properties may be lost due to pellicle formation. Secondly, structures limited to the outer surface may not be sustainable in the oral cavity since they will be lost over time due to erosion and degradation of the outermost surface. Although still relatively limited data are available yet, smooth biphasic nano- and microtextures may be very promising in the future. Especially resin-based composites or solid materials with incorporated nano- or microsized insertions combine several favorable features. Used alternations might be polar–nonpolar, positively–negatively charged, or hard–soft physicochemical properties. Many bacteria can adopt and adhere to interfaces with very different properties, but it is not clarified how well they can adapt to substrates that change their properties across a presumable adhesion area (Fig. 7a). Total vanishing of the synthetic identity of the alternating surface properties by adsorption of biomolecules is most likely a priori prevented, because of translation into a biologically alternating identity due to modulated adsorption intensities (Fig. 7b), alternating molecular conformations, or differently extended hard and soft protein coronas on, for example, surface partitions with low and high energies. Not at least, nanotextures might induce adsorption of loosely bound protein layer, which will delaminate or can easily be removed when reaching a certain thickness or a destabilizing load with microbes (Fig. 7c). Such surfaces would combine anti-adhesive properties with presumable good biocompatibility, and when applied with insertions, antirepellent properties are not limited to the surface, thereby tolerating certain degrees of biodegradation.

## Conclusions

Advances in the design and quality of artificial prosthetic materials in the oral environment and other parts of the body vastly increased the life span and life quality of people. But as outlined, still many problems arise with the control of microbial settlement and propagation on these surfaces. Therefore, it is a worthwhile and challenging endeavor to invest in the improvement of these materials to reduce complications and recurrence of oral and other diseases. In particular, there are many possibilities to improve existing prosthetic or filling materials by nanostructuring or functionalization of surfaces. In addition, combinatorial approaches of several strategies may lead to vastly improved materials for use in the oral environment. However, most promising for the future may be functionalized biphasic structures by incorporation of nanoparticles into existing dental materials due to presumably high functionality, durability, and biocompatibility.

In summary, novel enhanced materials and resin-based composites for coating of dental restorations, fillings, fixed particles, dentures, and prosthetic implants or orthodontic devices will hopefully lead to less complications or failures of these materials, thereby reducing the risk of caries, periodontitis, gingivitis, peri-implantitis, or other implant-related diseases.

## References

[1] Lamont RJ, Koo H, Hajishengallis G (2018). The oral microbiota: dynamic communities and host interactions. Nat Rev Microbiol.

[2] Dewhirst FE, Chen T, Izard J (2010). The human oral microbiome. J Bacteriol.

[3] Rupf S, Laczny CC, Galata V (2018). Comparison of initial oral microbiomes of young adults with and without cavitated dentin caries lesions using an in situ biofilm model. Sci Rep.

[4] Bowen WH, Burne RA, Wu H, Koo H (2019). Oral biofilms: pathogens, matrix and polymicrobial interactions in microenvironments. Trends Microbiol.

[5] Mark Welch JL, Dewhirst FE, Borisy GG (2019). Biogeography of the oral microbiome: the site-specialist hypothesis. Annu Rev Microbiol.

[6] He X, McLean JS, Guo L (2014). The social structure of microbial community involved in colonization resistance. ISME J.

[7] Sorbara MT, Pamer EG (2019). Interbacterial mechanisms of colonization resistance and the strategies pathogens use to overcome them. Mucosal Immunol.

[8] Graves DT, Corrêa JD, Silva TA (2019). The oral microbiota is modified by systemic diseases. J Dent Res.

[9] Idris A, Hasnain SZ, Huat LZ, Koh D (2017). Human diseases, immunity and the oral microbiota—insights gained from metagenomic studies. Oral Sci Int.

[10] Pitts NB, Zero DT, Marsh PD (2017). Dental caries. Nat Rev Dis Primers.

[11] Dahlen G, Basic A, Bylund J (2019). Importance of virulence factors for the persistence of oral bacteria in the inflamed gingival crevice and in the pathogenesis of periodontal disease. J Clin Med.

[12] Kurgan S, Kantarci A (2018). Molecular basis for immunohistochemical and inflammatory changes during progression of gingivitis to periodontitis. Periodontol 2000.

[13] Popova C, Dosseva-Panova V, Panov V (2013). Microbiology of periodontal diseases. A review. Biotechnol Biotechnol Equip.

[14] Nowicki EM, Shroff R, Singleton JA (2018). Microbiota and metatranscriptome changes accompanying the onset of gingivitis. MBio.

[15] Shi M, Wei Y, Hu W (2018). The subgingival microbiome of periodontal pockets with different probing depths in chronic and aggressive periodontitis: a pilot study. Front Cell Infect Microbiol.

[16] Hajishengallis G, Lamont RJ (2014). Breaking bad: manipulation of the host response by Porphyromonas gingivalis. Eur J Immunol.

[17] Hajishengallis G, Lamont RJ (2012). Beyond the red complex and into more complexity: the polymicrobial synergy and dysbiosis (PSD) model of periodontal disease etiology. Mol Oral Microbiol.

[18] Deng ZL, Szafrański SP, Jarek M (2017). Dysbiosis in chronic periodontitis: key microbial players and interactions with the human host. Sci Rep.

[19] Topcuoglu N, Kulekci G (2015). 16S rRNA based microarray analysis of ten periodontal bacteria in patients with different forms of periodontitis. Anaerobe.

[20] Smeets R, Henningsen A, Jung O (2014). Definition, etiology, prevention and treatment of peri-implantitis - a review. Head Face Med.

[21] Askar H, Krois J, Göstemeyer G (2020). Secondary caries: what is it, and how it can be controlled, detected, and managed?. Clin Oral Investig.

[22] Kim J (2018). Mathematical modeling approaches to describe the dynamics of protein adsorption at solid interfaces. Colloids Surfaces B Biointerfaces.

[23] Langmuir I (1918). The adsorption of gases on plane surfaces of glass, mica and platinum. J Am Chem Soc.

[24] Schaaf P, Talbot J (1989). Surface exclusion effects in adsorption processes. J Chem Phys.

[25] Faccio G (2018). From protein features to sensing surfaces. Sensors (Switzerland).

[26] Marsh JA, Teichmann SA (2011). Relative solvent accessible surface area predicts protein conformational changes upon binding. Structure.

[27] Roach P, Farrar D, Perry CC (2005). Interpretation of protein adsorption: surface-induced conformational changes. J Am Chem Soc.

[28] Magyari K, Vanea E, Baia L, Simon V (2016). Attachment and conformational changes of collagen on bioactive glass surface. Biomed Mater Eng.

[29] Sanfeld A, Royer C, Steinchen A (2015). Thermodynamic, kinetic and conformational analysis of proteins diffusion–sorption on a solid surface. Adv Colloid Interface Sci.

[30] Hirsh SL, McKenzie DR, Nosworthy NJ (2013). The Vroman effect: competitive protein exchange with dynamic multilayer protein aggregates. Colloids Surfaces B Biointerfaces.

[31] Horbett TA (2018). Fibrinogen adsorption to biomaterials. J Biomed Mater Res A.

[32] Keskin O, Tuncbag N, Gursoy A (2016). Predicting protein-protein interactions from the molecular to the proteome level. Chem Rev.

[33] Zhou HX, Pang X (2018). Electrostatic interactions in protein structure, folding, binding, and condensation. Chem Rev.

[34] Mulheran PA, Connell DJ, Kubiak-Ossowska K (2016). Steering protein adsorption at charged surfaces: electric fields and ionic screening. RSC Adv.

[35] Rabe M, Verdes D, Seeger S (2011). Understanding protein adsorption phenomena at solid surfaces. Adv Colloid Interface Sci.

[36] Falde EJ, Yohe ST, Colson YL, Grinstaff MW (2016). Superhydrophobic materials for biomedical applications. Biomaterials.

[37] Migliorini E, Weidenhaupt M, Picart C (2018). Practical guide to characterize biomolecule adsorption on solid surfaces (review). Biointerphases.

[38] Guo J, Yao X, Ning L (2014). The adsorption mechanism and induced conformational changes of three typical proteins with different secondary structural features on graphene. RSC Adv.

[39] Carniello V, Peterson BW, van der Mei HC, Busscher HJ (2018). Physico-chemistry from initial bacterial adhesion to surface-programmed biofilm growth. Adv Colloid Interface Sci.

[40] Hannig M, Joiner A (2006). The structure, function and properties of the acquired pellicle. Teeth Their Environ.

[41] Hannig C, Hannig M (2009). The oral cavity - a key system to understand substratum-dependent bioadhesion on solid surfaces in man. Clin Oral Investig.

[42] Hermansson M (1999). The DLVO theory in microbial adhesion. Colloids Surfaces B Biointerfaces.

[43] Van Der Westen R, Sjollema J, Molenaar R (2018). Floating and tether-coupled adhesion of bacteria to hydrophobic and hydrophilic surfaces. Langmuir.

[44] Teughels W, Van Assche N, Sliepen I, Quirynen M (2006). Effect of material characteristics and/or surface topography on biofilm development. Clin Oral Implants Res.

[45] Hori K, Matsumoto S (2010). Bacterial adhesion: from mechanism to control. Biochem Eng J.

[46] Spengler C, Thewes N, Jung P (2017). Determination of the nano-scaled contact area of staphylococcal cells. Nanoscale.

[47] Thewes N, Loskill P, Jung P (2014). Hydrophobic interaction governs unspecific adhesion of staphylococci: a single cell force spectroscopy study. Beilstein J Nanotechnol.

[48] Sjollema J, Van Der Mei HC, Hall CL (2017). Detachment and successive re-attachment of multiple, reversibly-binding tethers result in irreversible bacterial adhesion to surfaces. Sci Rep.

[49] Chen Y, Harapanahalli AK, Busscher HJ (2014). Nanoscale cell wall deformation impacts long-range bacterial adhesion forces on surfaces. Appl Environ Microbiol.

[50] Harapanahalli AK, Chen Y, Li J (2015). Influence of adhesion force on icaA and cidA gene expression and production of matrix components in Staphylococcus aureus biofilms. Appl Environ Microbiol.

[51] Wang C, Hou J, van der Mei HC et al (2019) Emergent properties in Streptococcus mutans biofilms are controlled through adhesion force sensing by initial colonizers. MBio 10:e1908–e19018. 10.1128/mbio.01908-19 10.1128/mBio.01908-19 PMC6737243 31506311

[52] Ren Y, Wang C, Chen Z (2018). Emergent heterogeneous microenvironments in biofilms: substratum surface heterogeneity and bacterial adhesion force-sensing. FEMS Microbiol Rev.

[53] Kim J, Kim HS, Han S (2013). Hydrodynamic effects on bacterial biofilm development in a microfluidic environment. Lab Chip.

[54] Thomen P, Robert J, Monmeyran A (2017). Bacterial biofilm under flow: first a physical struggle to stay, then a matter of breathing. PLoS One.

[55] Wenzel RN (1936). Resistance of solid surfaces to wetting by water. Ind Eng Chem.

[56] Simpson JT, Hunter SR, Aytug T (2015). Superhydrophobic materials and coatings: a review. Reports Prog Phys.

[57] Yang C, Tartaglino U, Persson BNJ (2008). Nanodroplets on rough hydrophilic and hydrophobic surfaces. Eur Phys J E.

[58] Cassie ABD, Baxter S (1944). Wettability of porous surfaces. Trans Faraday Soc.

[59] Zhang X, Wang L, Levänen E (2013). Superhydrophobic surfaces for the reduction of bacterial adhesion. RSC Adv.

[60] Hannig M, Hannig C, Lussi A, Ganss C (2014). The pellicle and erosion. Erosive tooth wear: from diagnosis to therapy.

[61] Siqueira WL, Custodio W, McDonald EE (2012). New insights into the composition and functions of the acquired enamel pellicle. J Dent Res.

[62] Hannig M (1999). Ultrastructural investigation of pellicle morphogenesis at two different intraoral sites during a 24-h period. Clin Oral Investig.

[63] Güth-Thiel S, Kraus-Kuleszka I, Mantz H (2019). Comprehensive measurements of salivary pellicle thickness formed at different intraoral sites on Si wafers and bovine enamel. Colloids Surfaces B Biointerfaces.

[64] Schweigel H, Wicht M, Schwendicke F (2016). Salivary and pellicle proteome: a datamining analysis. Sci Rep.

[65] Hannig M (1997). Transmission electron microscopic study of in vivo pellicle formation on dental restorative materials. Eur J Oral Sci.

[66] Cassiano LPS, Ventura TMS, Silva CMS (2018). Protein profile of the acquired enamel pellicle after rinsing with whole milk, fat-free milk, and water: an in vivo study. Caries Res.

[67] Trautmann S, Barghash A, Fecher-Trost C (2019). Proteomic analysis of the initial oral pellicle in caries-active and caries-free individuals. Proteomics Clin Appl.

[68] Delius J, Trautmann S, Médard G (2017). Label-free quantitative proteome analysis of the surface-bound salivary pellicle. Colloids Surfaces B Biointerfaces.

[69] Nobbs AH, Jenkinson HF, Jakubovics NS (2011). Stick to your gums: mechanisms of oral microbial adherence. J Dent Res.

[70] Cisar JO, Takahashi Y, Ruhl S (1997). Specific inhibitors of bacterial adhesion: observations from the study of gram-positive bacteria that initiate biofilm formation on the tooth surface. Adv Dent Res.

[71] Ruhl S, Sandberg AL, Cisar JO (2004). Salivary receptors for the proline-rich protein-binding and lectin-like adhesins of oral actinomyces and streptococci. J Dent Res.

[72] Kirsch J, Hannig C, Pötschke S (2017). Enzymology and ultrastructure of the in situ pellicle in caries-active and caries-inactive patients. Caries Res.

[73] Schulz A, Lang R, Behr J (2020). Targeted metabolomics of pellicle and saliva in children with different caries activity. Sci Rep.

[74] Milosevic A (1992). The influence of surface finish and in-vitro pellicle on contact-angle measurement and surface morphology of three commercially available composite restoratives. J Oral Rehabil.

[75] McConnell MD, Liu Y, Nowak AP (2010). Bacterial plaque retention on oral hard materials: effect of surface roughness, surface composition, and physisorbed polycarboxylate. J Biomed Mater Res A.

[76] Park JW, Song CW, Jung JH (2012). The effects of surface roughness of composite resin on biofilm formation of Streptococcus mutans in the presence of saliva. Oper Dent.

[77] Cavalcanti YW, Soare RV, Leite Assis MA (2015). Titanium surface roughing treatments contribute to higher interaction with salivary proteins MG2 and lactoferrin. J Contemp Dent Pract.

[78] Ionescu AC, Cazzaniga G, Ottobelli M (2018). In vitro biofilm formation on resin-based composites cured under different surface conditions. J Dent.

[79] Cheaib Z, Rakmathulina E, Lussi A, Eick S (2015). Impact of acquired pellicle modification on adhesion of early colonizers. Caries Res.

[80] Cavalcanti YW, Wilson M, Lewis M (2016). Salivary pellicles equalise surfaces’ charges and modulate the virulence of Candida albicans biofilm. Arch Oral Biol.

[81] Kline KA, Fälker S, Dahlberg S (2009). Bacterial adhesins in host-microbe interactions. Cell Host Microbe.

[82] Vengadesan K, Narayana SVL (2011). Structural biology of Gram-positive bacterial adhesins. Protein Sci.

[83] Hansmeier N, Miskiewicz K, Elpers L (2017). Functional expression of the entire adhesiome of Salmonella enterica serotype Typhimurium. Sci Rep.

[84] Sullan RMA, Li JK, Crowley PJ (2015). Binding forces of Streptococcus mutans P1 adhesin. ACS Nano.

[85] Liang X, Liu B, Zhu F (2016). A distinct sortase SrtB anchors and processes a streptococcal adhesin AbpA with a novel structural property. Sci Rep.

[86] Back CR, Sztukowska MN, Till M (2017). The Streptococcus gordonii adhesin CshA protein binds host fibronectin via a catch-clamp mechanism. J Biol Chem.

[87] Cross BW, Ruhl S (2018). Glycan recognition at the saliva – oral microbiome interface. Cell Immunol.

[88] Thamadilok S, Roche-Hakansson H, Hakansson AP, Ruhl S (2016). Absence of capsule reveals glycan-mediated binding and recognition of salivary mucin Muc7 by Streptococcus pneumoniae. Mol Oral Microbiol.

[89] Avilés-Reyes A, Miller JH, Lemos JA, Abranches J (2017). Collagen-binding proteins of Streptococcus mutans and related streptococci. Mol Oral Microbiol.

[90] Nomura R, Ogaya Y, Nakano K (2016). Contribution of the collagen-binding proteins of Streptococcus mutans to bacterial colonization of inflamed dental pulp. PLoS One.

[91] Heller D, Helmerhorst EJ, Gower AC (2016). Microbial diversity in the early in vivo-formed dental biofilm. Appl Environ Microbiol.

[92] Espinoza JL, Harkins DM, Torralba M (2018). Supragingival plaque microbiome ecology and functional potential in the context of health and disease. MBio.

[93] Koo H, Falsetta ML, Klein MI (2013). The exopolysaccharide matrix: a virulence determinant of cariogenic biofilm. J Dent Res.

[94] Palmer RJ, Shah N, Valm A (2017). Interbacterial adhesion networks within early oral biofilms of single human hosts. Appl Environ Microbiol.

[95] Wen ZT, Yates D, Ahn SJ, Burne RA (2010) Biofilm formation and virulence expression by Streptococcus mutans are altered when grown in dual-species model. BMC Microbiol:10. 10.1186/1471-2180-10-111 10.1186/1471-2180-10-111 PMC2867949 20398271

[96] Shemesh M, Tam A, Aharoni R, Steinberg D (2010). Genetic adaptation of Streptococcus mutans during biofilm formation on different types of surfaces. BMC Microbiol.

[97] Diaz PI, Dupuy AK, Abusleme L (2012). Using high throughput sequencing to explore the biodiversity in oral bacterial communities. Mol Oral Microbiol.

[98] Segata N, Haake SK, Mannon P (2012). Composition of the adult digestive tract bacterial microbiome based on seven mouth surfaces, tonsils, throat and stool samples. Genome Biol.

[99] Eren AM, Borisy GG, Huse SM, Mark Welch JL (2014). Oligotyping analysis of the human oral microbiome. Proc Natl Acad Sci U S A.

[100] Lempel E, Tóth Á, Fábián T (2015). Retrospective evaluation of posterior direct composite restorations: 10-year findings. Dent Mater.

[101] Nedeljkovic I, Teughels W, De Munck J (2015). Is secondary caries with composites a material-based problem?. Dent Mater.

[102] Imazato S (2003). Antibacterial properties of resin composites and dentin bonding systems. Dent Mater.

[103] Leonhardt OJ, Dahlén G (1995). Bacterial colonization on titanium, hydroxyapatite, and amalgam surfaces in vivo. J Dent Res.

[104] Bourbia M, Ma D, Cvitkovitch DG (2013). Cariogenic bacteria degrade dental resin composites and adhesives. J Dent Res.

[105] Zhang N, Melo MAS, Weir MD (2016). Do dental resin composites accumulate more oral biofilms and plaque than amalgam and glass ionomer materials?. Materials (Basel).

[106] Sousa RP, Zanin ICJ, Lima JPM (2009). In situ effects of restorative materials on dental biofilm and enamel demineralisation. J Dent.

[107] Mo S, Bao W, Lai G (2010). The microfloral analysis of secondary caries biofilm around class I and class II composite and amalgam fillings. BMC Infect Dis.

[108] Padovani GC, Fùcio SBP, Ambrosano GMB (2015). In situ bacterial accumulation on dental restorative materials. CLSM/COMSTAT analysis. Am J Dent.

[109] Conrads G, Wendt LK, Hetrodt F (2019). Deep sequencing of biofilm microbiomes on dental composite materials. J Oral Microbiol.

[110] Chau NPT, Pandit S, Jung J-E (2016). Long-term anti-cariogenic biofilm activity of glass ionomers related to fluoride release. J Dent.

[111] Mayanagi G, Igarashi K, Washio J (2014). Effect of fluoride-releasing restorative materials on bacteria-induced pH fall at the bacteria–material interface: an in vitro model study. J Dent.

[112] Miki S, Kitagawa H, Kitagawa R (2016). Antibacterial activity of resin composites containing surface pre-reacted glass-ionomer (S-PRG) filler. Dent Mater.

[113] Wang S, Guo L, Seneviratne CJ (2014). Biofilm formation of salivary microbiota on dental restorative materials analyzed by denaturing gradient gel electrophoresis and sequencing. Dent Mater J.

[114] Ma S, Imazato S, Chen JH (2012). Effects of a coating resin containing S-PRG filler to prevent demineralization of root surfaces. Dent Mater J.

[115] Kim KH, Loch C, Waddell JN et al (2017) Surface characteristics and biofilm development on selected dental ceramic materials. Int J Dent:2017. 10.1155/2017/7627945 10.1155/2017/7627945 PMC5439072 28567055

[116] Rashid H (2014). The effect of surface roughness on ceramics used in dentistry: a review of literature. Eur J Dent.

[117] Bremer F, Grade S, Kohorst P, Stiesch M (2011). In vivo biofilm formation on different dental ceramics. Quintessence Int.

[118] Goodson JM, Shoher I, Imber S (2001). Reduced dental plaque accumulation on composite gold alloy margins. J Periodontal Res.

[119] Auschill TM, Arweiler NB, Brecx M (2002). The effect of dental restorative materials on dental biofilm. Eur J Oral Sci.

[120] Ismail F, Eisenburger M, Grade S, Stiesch M (2016). In situ biofilm formation on titanium, gold alloy and zirconia abutment materials. Dentistry.

[121] Dittmer MP, Hellemann CF, Grade S (2015). Comparative three-dimensional analysis of initial biofilm formation on three orthodontic bracket materials. Head Face Med.

[122] Urushibara Y, Ohshima T, Sato M (2014). An analysis of the biofilms adhered to framework alloys using in vitro denture plaque models. Dent Mater J.

[123] Jordan RPC, Marsh L, Ayre WN (2016). An assessment of early colonisation of implant-abutment metal surfaces by single species and co-cultured bacterial periodontal pathogens. J Dent.

[124] Souza JCM, Mota RRC, Sordi MB (2016). Biofilm formation on different materials used in oral rehabilitation. Braz Dent J.

[125] Gleiznys A, Zdanavičienė E, Žilinskas J (2015). Candida albicans importance to denture wearers. A literature review. Stomatologija.

[126] Morse DJ, Smith A, Wilson MJ (2019). Molecular community profiling of the bacterial microbiota associated with denture-related stomatitis. Sci Rep.

[127] O’Donnell LE, Alalwan HKA, Kean R (2017). Candida albicans biofilm heterogeneity does not influence denture stomatitis but strongly influences denture cleansing capacity. J Med Microbiol.

[128] Korsch M, Walther W, Marten SM, Obst U (2014). Microbial analysis of biofilms on cement surfaces: an investigation in cement-associated peri-implantitis. J Appl Biomater Fundam Mater.

[129] Korsch M, Marten SM, Dötsch A (2016). Effect of dental cements on peri-implant microbial community: comparison of the microbial communities inhabiting the peri-implant tissue when using different luting cements. Clin Oral Implants Res.

[130] Korsch M, Marten SM, Walther W (2018). Impact of dental cement on the peri-implant biofilm-microbial comparison of two different cements in an in vivo observational study. Clin Implant Dent Relat Res.

[131] Cortes-Acha B, Figueiredo R, Blanc V (2019). Development and viability of biofilms grown on experimental abutments mimicking dental implants: an in vivo model. Med Oral Patol Oral y Cir Bucal.

[132] Cortés-Acha B, Figueiredo R, Seminago R (2017). Microbiota analysis of biofilms on experimental abutments mimicking dental implants: an in vivo model. J Periodontol.

[133] Salvi GE, Cosgarea R, Sculean A (2017). Prevalence and mechanisms of peri-implant diseases. J Dent Res.

[134] Kumar PS, Mason MR, Brooker MR, O’Brien K (2012). Pyrosequencing reveals unique microbial signatures associated with healthy and failing dental implants. J Clin Periodontol.

[135] Zheng H, Xu L, Wang Z (2015). Subgingival microbiome in patients with healthy and ailing dental implants. Sci Rep.

[136] Sanz-Martin I, Doolittle-Hall J, Teles RP (2017). Exploring the microbiome of healthy and diseased peri-implant sites using Illumina sequencing. J Clin Periodontol.

[137] Shibli JA, Melo L, Ferrari DS (2008). Composition of supra- and subgingival biofilm of subjects with healthy and diseased implants. Clin Oral Implants Res.

[138] do Nascimento C, Pita MS, de Souza Santos E (2016). Microbiome of titanium and zirconia dental implants abutments. Dent Mater.

[139] Do Nascimento C, Pita MS, Pedrazzi V (2013). In vivo evaluation of Candida spp. adhesion on titanium or zirconia abutment surfaces. Arch Oral Biol.

[140] Raffaini FC, Freitas AR, Silva TSO (2018). Genome analysis and clinical implications of the bacterial communities in early biofilm formation on dental implants restored with titanium or zirconia abutments. Biofouling.

[141] do Nascimento C, Pita MS, Nogueira FH (2013). Bacterial adhesion on the titanium and zirconia abutment surfaces. Clin Oral Implants Res.

[142] Größner-Schreiber B, Teichmann J, Hannig M (2009). Modified implant surfaces show different biofilm compositions under in vivo conditions. Clin Oral Implants Res.

[143] de Freitas AR, de O Silva TS, Ribeiro RF (2018). Oral bacterial colonization on dental implants restored with titanium or zirconia abutments: 6-month follow-up. Clin Oral Investig.

[144] Hao Y, Huang X, Zhou X (2018). Influence of dental prosthesis and restorative materials interface on oral biofilms. Int J Mol Sci.

[145] Souza JGS, Costa Oliveira BE, Bertolini M (2019). Titanium particles and ions favor dysbiosis in oral biofilms. J Periodont Res.

[146] Fretwurst T, Nelson K, Tarnow DP (2018). Is metal particle release associated with peri-implant bone destruction? An emerging concept. J Dent Res.

[147] Hannig M (1999). Transmission electron microscopy of early plaque formation on dental materials in vivo. Eur J Oral Sci.

[148] Lima EMCX, Koo H, Vacca Smith AM (2008). Adsorption of salivary and serum proteins, and bacterial adherence on titanium and zirconia ceramic surfaces. Clin Oral Implants Res.

[149] Hannig C, Wasser M, Becker K (2006). Influence of different restorative materials on lysozyme and amylase activity of the salivary pellicle in situ. J Biomed Mater Res A.

[150] Hannig C, Huber K, Lambrichts I (2007). Detection of salivary alpha-amylase and lysozyme exposed on the pellicle formed in situ on different materials. J Biomed Mater Res A.

[151] Yoshida E, Hayakawa T (2013). Adsorption study of pellicle proteins to gold, silica and titanium by quartz crystal microbalance method. Dent Mater J.

[152] Pantaroto HN, Amorim KP, Matozinho Cordeiro J (2019). Proteome analysis of the salivary pellicle formed on titanium alloys containing niobium and zirconium. Biofouling.

[153] Thomas RZ, Van Der Mei HC, Van Der Veen MH (2008). Bacterial composition and red fluorescence of plaque in relation to primary and secondary caries next to composite: an in situ study. Oral Microbiol Immunol.

[154] Pogodin S, Hasan J, Baulin VA (2013). Biophysical model of bacterial cell interactions with nanopatterned cicada wing surfaces. Biophys J.

[155] Hannig M, Kriener L, Hoth-Hannig W (2007). Influence of nanocomposite surface coating on biofilm formation in situ. J Nanosci Nanotechnol.

[156] Jenny N, Naorem S, Naorem K, Singh PD (2017). Know about biocompatibility of dental materials : a review. Pyrex J Med Med Sci.

[157] Monsees TK (2016). Biocompatibility and anti-microbiological activity characterization of novel coatings for dental implants: a primer for non-biologists. Front Mater.

[158] Wataha JC, Lockwood PE (1998). Release of elements from dental casting alloys into cell-culture medium over 10 months. Dent Mater.

[159] Elshahawy W, Watanabe I (2014). Biocompatibility of dental alloys used in dental fixed prosthodontics. Tanta Dent J.

[160] Sjogren G, Sletten G, DJE (2000). Cytotoxicity of dental alloys, metals, and ceramics assessed by Millipore. J Prosthet Dent.

[161] Yang HC, Pon LA (2003). Toxicity of metal ions used in dental alloys: a study in the yeast Saccharomyces cerevisiae. Drug Chem Toxicol.

[162] Fathi M, Mortazavi V (2004). A review on dental amalgam corrosion and its consequences. J Res Med Sci.

[163] Freire WP, Fook MVL, Barbosa EF (2015). Biocompatibility of dental restorative materials. Mater Sci Forum.

[164] Sidhu S, Nicholson J (2016). A review of glass-ionomer cements for clinical dentistry. J Funct Biomater.

[165] Priyadarsini S, Mukherjee S, Mishra M (2018). Nanoparticles used in dentistry: a review. J Oral Biol Craniofacial Res.

[166] Wang J, Wang L, Fan Y (2016). Adverse biological effect of TiO2 and hydroxyapatite nanoparticles used in bone repair and replacement. Int J Mol Sci.

[167] Choi C-H, Hagvall SH, Wu BM (2007). Cell interaction with three-dimensional sharp-tip nanotopography. Biomaterials.

[168] Jang Y, Choi WT, Johnson CT (2018). Inhibition of bacterial adhesion on nanotextured stainless steel 316L by electrochemical etching. ACS Biomater Sci Eng.

[169] Cheng Y, Feng G, Moraru CI (2019). Micro- and nanotopography sensitive bacterial attachment mechanisms: a review. Front Microbiol.

[170] Quirynen M, Bollen CML (1995). The influence of surface roughness and surface-free energy on supra- and subgingival plaque formation in man. J Clin Periodontol.

[171] Fröjd V, Chávez de Paz L, Andersson M (2011). In situ analysis of multispecies biofilm formation on customized titanium surfaces. Mol Oral Microbiol.

[172] Xing R, Lyngstadaas SP, Ellingsen JE (2015). The influence of surface nanoroughness, texture and chemistry of TiZr implant abutment on oral biofilm accumulation. Clin Oral Implants Res.

[173] Bürgers R, Gerlach T, Hahnel S (2010). In vivo and in vitro biofilm formation on two different titanium implant surfaces. Clin Oral Implants Res.

[174] Al-Ahmad A, Wiedmann-Al-Ahmad M, Faust J (2010). Biofilm formation and composition on different implant materials in vivo. J Biomed Mater Res B Appl Biomater.

[175] de Melo F, do Nascimento C, Souza DO, de Albuquerque RF (2017). Identification of oral bacteria on titanium implant surfaces by 16S rDNA sequencing. Clin Oral Implants Res.

[176] Ribeiro CF, Cogo-Müller K, Franco GC (2016). Initial oral biofilm formation on titanium implants with different surface treatments: an in vivo study. Arch Oral Biol.

[177] Conserva E, Generali L, Bandieri A (2018). Plaque accumulation on titanium disks with different surface treatments: an in vivo investigation. Odontology.

[178] Al-Ahmad A, Karygianni L, Wartenhorst MS (2016). Bacterial adhesion and biofilm formation on yttriastabilized, tetragonal zirconia and titanium oral implant materials with low surface roughness - an in situ study. J Med Microbiol.

[179] Bevilacqua L, Milan A, Del Lupo V (2018). Biofilms developed on dental implant titanium surfaces with different roughness: comparison between in vitro and in vivo studies. Curr Microbiol.

[180] Bollen CM, Lambrechts P, Quirynen M (1997). Comparison of surface roughness of oral hard materials to the threshold surface roughness for bacterial plaque retention: a review of the literature. Dent Mater.

[181] Quirynen M, Bollen CM, Papaioannou W (1996). The influence of titanium abutment surface roughness on plaque accumulation and gingivitis: short-term observations. Int J Oral Maxillofac Implants.

[182] Taha M, El-Fallal A, Degla H (2016). In vitro and in vivo biofilm adhesion to esthetic coated arch wires and its correlation with surface roughness. Angle Orthod.

[183] Gadelmawla ES, Koura MM, Maksoud TMA (2002). Roughness parameters. J Mater Process Technol.

[184] Jumelle C, Hamri A, Egaud G (2017). Comparison of four methods of surface roughness assessment of corneal stromal bed after lamellar cutting. Biomed Opt Express.

[185] Duparré A, Ferre-Borrull J, Gliech S (2002). Surface characterization techniques for determining the root-mean-square roughness and power spectral densities of optical components. Appl Opt.

[186] Young PL, Brackbill TP, Kandlikar SG (2009). Comparison of roughness parameters for various microchannel surfaces in single-phase flow applications. Heat Transf Eng.

[187] Rizzello L, Galeone A, Vecchio G (2012). Molecular response of Escherichia coli adhering onto nanoscale topography. Nanoscale Res Lett.

[188] Rizzello L, Sorce B, Sabella S (2011). Impact of nanoscale topography on genomics and proteomics of adherent bacteria. ACS Nano.

[189] Rzhepishevska O, Hakobyan S, Ruhal R (2013). The surface charge of anti-bacterial coatings alters motility and biofilm architecture. Biomater Sci.

[190] Kiremitci-Gumusderelioglu M, Peşmen A (1996). Microbial adhesion to ionogenic PHEMA, PU PP implants. Biomaterials.

[191] Yuan Y, Hays MP, Hardwidge PR, Kim J (2017). Surface characteristics influencing bacterial adhesion to polymeric substrates. RSC Adv.

[192] Wassmann T, Kreis S, Behr M, Buergers R (2017) The influence of surface texture and wettability on initial bacterial adhesion on titanium and zirconium oxide dental implants. Int J Implant Dent 3. 10.1186/s40729-017-0093-3 10.1186/s40729-017-0093-3 PMC5511811 28714053

[193] Dou XQ, Zhang D, Feng C, Jiang L (2015). Bioinspired hierarchical surface structures with tunable wettability for regulating bacteria adhesion. ACS Nano.

[194] Oliveira AS, Kaizer MR, Azevedo MS (2015). (Super)hydrophobic coating of orthodontic dental devices and reduction of early oral biofilm retention. Biomed Mater.

[195] Elter C, Heuer W, Demling A (2011). Comparative analysis of biofilm formation on dental implant abutments with respect to supra- and subgingival areas: polytetrafluoroethylene versus titanium. Int J Prosthodont.

[196] Demling A, Elter C, Heidenblut T (2010). Reduction of biofilm on orthodontic brackets with the use of a polytetrafluoroethylene coating. Eur J Orthod.

[197] Ensikat HJ, Ditsche-Kuru P, Neinhuis C, Barthlott W (2011). Superhydrophobicity in perfection: the outstanding properties of the lotus leaf. Beilstein J Nanotechnol.

[198] Cheng YT, Rodak DE, Wong CA, Hayden CA (2006). Effects of micro- and nano-structures on the self-cleaning behaviour of lotus leaves. Nanotechnology.

[199] Hizal F, Rungraeng N, Lee J (2017). Nanoengineered superhydrophobic surfaces of aluminum with extremely low bacterial adhesivity. ACS Appl Mater Interfaces.

[200] Morán G, Ramos-Chagas G, Hugelier S (2018). Superhydrophobic polypyrene films to prevent: Staphylococcus aureus and Pseudomonas aeruginosa biofilm adhesion on surfaces: high efficiency deciphered by fluorescence microscopy. Photochem Photobiol Sci.

[201] Lee M, Kwon J, Jiang HB (2019). The antibacterial effect of non-thermal atmospheric pressure plasma treatment of titanium surfaces according to the bacterial wall structure. Sci Rep.

[202] Hwang GB, Page K, Patir A (2018). The anti-biofouling properties of superhydrophobic surfaces are short-lived. ACS Nano.

[203] Sousa C, Rodrigues D, Oliveira R (2011). Superhydrophobic poly(L-lactic acid) surface as potential bacterial colonization substrate. AMB Express.

[204] Covarrubias C, Trepiana D, Corral C (2018). Synthesis of hybrid copper-chitosan nanoparticles with antibacterial activity against cariogenic Streptococcus mutans. Dent Mater J.

[205] Farhoudian S, Yadollahi M, Namazi H (2016). Facile synthesis of antibacterial chitosan/CuO bio-nanocomposite hydrogel beads. Int J Biol Macromol.

[206] Sanpui P, Murugadoss A, Prasad PVD (2008). The antibacterial properties of a novel chitosan–Ag-nanoparticle composite. Int J Food Microbiol.

[207] González J, Covarrubias C, Cádiz M (2016). Design of antimicrobial release systems based on chitosan and copper nanoparticles for localized periodontal therapy. J Dent Oral Disord.

[208] Memarzadeh K, Sharili AS, Huang J (2015). Nanoparticulate zinc oxide as a coating material for orthopedic and dental implants. J Biomed Mater Res A.

[209] Arakha M, Pal S, Samantarrai D (2015). Antimicrobial activity of iron oxide nanoparticle upon modulation of nanoparticle-bacteria interface. Sci Rep.

[210] Stankic S, Suman S, Haque F, Vidic J (2016). Pure and multi metal oxide nanoparticles: synthesis, antibacterial and cytotoxic properties. J Nanobiotechnology.

[211] Chatzistavrou X, Lefkelidou A, Papadopoulou L (2018). Bactericidal and bioactive dental composites. Front Physiol.

[212] Jin J, Zhang L, Shi M (2017). Ti-GO-Ag nanocomposite: the effect of content level on the antimicrobial activity and cytotoxicity. Int J Nanomed.

[213] Mangal U, Kim J-Y, Seo J-Y (2019). Novel poly (methyl methacrylate) containing nanodiamond to improve the mechanical properties and fungal resistance. Materials (Basel).

[214] Acosta LD, Pérez-Camacho O, Acosta R et al (2019) Reduction of Candida albicans biofilm formation by coating polymethyl methacrylate denture bases with a photopolymerized film. J Prosthet Dent. 10.1016/j.prosdent.2019.08.003 10.1016/j.prosdent.2019.08.003 31831165

[215] Zhang Y, Chen YY, Huang L (2017). The antifungal effects and mechanical properties of silver bromide/cationic polymer nano-composite-modified poly-methyl methacrylate-based dental resin. Sci Rep.

[216] Feng J, Cheng L, Zhou X (2015). In situ antibiofilm effect of glass-ionomer cement containing dimethylaminododecyl methacrylate. Dent Mater.

[217] Feng J, Cheng L, Zhou X (2019). Effects of water aging on the mechanical and anti-biofilm properties of glass-ionomer cement containing dimethylaminododecyl methacrylate. Dent Mater.

[218] Duque C, Aida KL, Pereira JA (2017). glass-ionomer cement containing chlorhexidine for atraumatic restorative treatment. J Appl Oral Sci.

[219] Frencken JE, Imazato S, Toi C (2007). Antibacterial effect of chlorhexidine-containing glass ionomer cement in vivo: a pilot study. Caries Res.

[220] Rupf S, Balkenhol M, Sahrhage TO (2012). Biofilm inhibition by an experimental dental resin composite containing octenidine dihydrochloride. Dent Mater.

[221] Mellegård H, Strand SP, Christensen BE (2011). Antibacterial activity of chemically defined chitosans: influence of molecular weight, degree of acetylation and test organism. Int J Food Microbiol.

[222] Stenhagen ISR, Rukke HV, Dragland IS, Kopperud HM (2019). Effect of methacrylated chitosan incorporated in experimental composite and adhesive on mechanical properties and biofilm formation. Eur J Oral Sci.

[223] Mahmoud MY, Steinbach-Rankins JM, Demuth DR (2019). Functional assessment of peptide-modified PLGA nanoparticles against oral biofilms in a murine model of periodontitis. J Control Release.

[224] Mahmoud MY, Demuth DR, Steinbach-Rankins JM (2018). BAR-encapsulated nanoparticles for the inhibition and disruption of Porphyromonas gingivalis-Streptococcus gordonii biofilms. J Nanobiotechnology.

[225] Xu LC, Wo Y, Meyerhoff ME, Siedlecki CA (2017). Inhibition of bacterial adhesion and biofilm formation by dual functional textured and nitric oxide releasing surfaces. Acta Biomater.

[226] Sundheim G, Langsrud S, Heir E, Holck AL (1998). Bacterial resistance to disinfectants containing quaternary ammonium compounds. Int Biodeterior Biodegrad.

[227] Kwaśniewska D, Chen YL, Wieczorek D (2020). Biological activity of quaternary ammonium salts and their derivatives. Pathogens.

[228] Whitehead KA, Colligon J, Verran J (2005). Retention of microbial cells in substratum surface features of micrometer and sub-micrometer dimensions. Colloids Surfaces B Biointerfaces.

[229] Helbig R, Günther D, Friedrichs J (2016). The impact of structure dimensions on initial bacterial adhesion. Biomater Sci.

[230] Hochbaum AI, Aizenberg J (2010). Bacteria pattern spontaneously on periodic nanostructure arrays. Nano Lett.

[231] Valle J, Burgui S, Langheinrich D (2015). Evaluation of surface microtopography engineered by direct laser interference for bacterial anti-biofouling. Macromol Biosci.

[232] Roessler F, Lasagni AF (2018). Protecting sub-micrometer surface features in polymers from mechanical damage using hierarchical patterns. J Laser Micro Nanoeng.

[233] Jung YC, Bhushan B (2009). Mechanically durable carbon nanotube - composite hierarchical structures with superhydrophobicity, self-cleaning, and low-drag. ACS Nano.

[234] Groten J, Rühe J (2013). Surfaces with combined microscale and nanoscale structures: a route to mechanically stable superhydrophobic surfaces?. Langmuir.

[235] Tebbs SE, Sawyer A, Elliott TS (1994). Influence of surface morphology on in vitro bacterial adherence to central venous catheters. Br J Anaesth.

[236] Flint SH, Brooks JD, Bremer PJ (2000). Properties of the stainless steel substrate, influencing the adhesion of thermo-resistant streptococci. J Food Eng.

[237] Díaz C, Schilardi PL, Salvarezza RC, de Mele MFL (2007). Nano/microscale order affects the early stages of biofilm formation on metal surfaces. Langmuir.

[238] Verran J, Packer A, Kelly P, Whitehead KA (2010). The retention of bacteria on hygienic surfaces presenting scratches of microbial dimensions. Lett Appl Microbiol.

[239] Ihnen AC, Lee J-H, Lee WY (2010). Effects of disordered hemispherical micropatterns on Staphylococcus epidermidis biofilm formation. Colloids Surf B Biointerfaces.

[240] Wu Y, Zitelli JP, TenHuisen KS (2011). Differential response of staphylococci and osteoblasts to varying titanium surface roughness. Biomaterials.

[241] Hannig C, Helbig R, Hilsenbeck J et al (2018) Impact of the springtail’s cuticle nanotopography on bioadhesion and biofilm formation in vitro and in the oral cavity. R Soc Open Sci 5. 10.1098/rsos.171742 10.1098/rsos.171742 PMC6083677 30109045

[242] Romano JM, Ahmed R, Garcia-Giron A (2019). Subwavelength direct laser nanopatterning via microparticle arrays for functionalizing metallic surfaces. J Micro Nano Manuf.

[243] Graham M, Cady N (2014). Nano and microscale topographies for the prevention of bacterial surface fouling. Coatings.

[244] Whitehead KA, Verran J (2006). The effect of surface topography on the retention of microorganisms. Trans IChemE Part C.

[245] Kerr A, Cowling MJ (2003). The effects of surface topography on the accumulation of biofouling. Philos Mag.

[246] Ivanova EP, Truong VK, Wang JY (2010). Impact of nanoscale roughness of titanium thin film surfaces on bacterial retention. Langmuir.

[247] Mitik-Dineva N, Wang J, Truong VK (2009). Escherichia coli, Pseudomonas aeruginosa, and Staphylococcus aureus attachment patterns on glass surfaces with nanoscale roughness. Curr Microbiol.

[248] Singh AV, Vyas V, Patil R (2011). Quantitative characterization of the influence of the nanoscale morphology of nanostructured surfaces on bacterial adhesion and biofilm formation. PLoS One.

[249] Satriano C, Messina GML, Carnazza S (2006). Bacterial adhesion onto nanopatterned polymer surfaces. Mater Sci Eng C Biomimetic Supramol Syst.

[250] Park MR, Banks MK, Applegate B, Webster TJ (2008). Influence of nanophase titania topography on bacterial attachment and metabolism. Int J Nanomed.

[251] Lundqvist M, Sethson I, Jonsson BH (2004). Protein adsorption onto silica nanoparticles: conformational changes depend on the particles’ curvature and the protein stability. Langmuir.

[252] Roach P, Farrar D, Perry CC (2006). Surface tailoring for controlled protein adsorption: effect of topography at the nanometer scale and chemistry. J Am Chem Soc.

[253] Anselme K, Davidson P, Popa AM (2010). The interaction of cells and bacteria with surfaces structured at the nanometre scale. Acta Biomater.

[254] Bazaka K, Crawford RJ, Ivanova EP (2011). Do bacteria differentiate between degrees of nanoscale surface roughness?. Biotechnol J.

[255] Lejars M, Margaillan A, Bressy C (2012). Fouling release coatings: a nontoxic alternative to biocidal antifouling coatings. Chem Rev.

[256] Chen S, Li L, Zhao C, Zheng J (2010). Surface hydration: principles and applications toward low-fouling/nonfouling biomaterials. Polymer (Guildf).

[257] Baier RE (2006). Surface behaviour of biomaterials: the theta surface for biocompatibility. J Mater Sci Mater Med.

[258] Epstein AK, Hochbaum AI, Kim P, Aizenberg J (2011). Control of bacterial biofilm growth on surfaces by nanostructural mechanics and geometry. Nanotechnology.

[259] Song F, Ren D (2014). Stiffness of cross-linked poly(dimethylsiloxane) affects bacterial adhesion and antibiotic susceptibility of attached cells. Langmuir.

[260] Beachey EH (1981). Bacterial adherence: adhesin-receptor interactions mediating the attachment of bacteria to mucosal surfaces. J Infect Dis.

[261] Gibbons RJ (1989). Bacterial adhesion to oral tissue: a model for infectious diseases. J Dent Res.

[262] Herrmann M, Vaudaux PE, Pittet D (1988). Fibronectin, fibrinogen, and laminin act as mediators of adherence of clinical staphylococcal isolates to foreign material. J Infect Dis.

[263] Fröman G, Switalski LM, Speziale P, Höök M (1987). Isolation and characterization of a fibronectin receptor from *Staphylococcus aureus*. J Biol Chem.

[264] Guégan C, Garderes J, Le Pennec G (2014). Alteration of bacterial adhesion induced by the substrate stiffness. Colloids Surfaces B Biointerfaces.

[265] Jaggessar A, Shahali H, Mathew A, Yarlagadda PKDV (2017). Bio-mimicking nano and micro-structured surface fabrication for antibacterial properties in medical implants. J Nanobiotechnol.

[266] McHale G, Newton MI, Shirtcliffe NJ (2009). Dynamic wetting and spreading and the role of topography. J Phys Condens Matter.

[267] Wagner T, Neinhuis C, Barthlott W (1996). Wettability and contaminability of insect wings as a function of their surface sculptures. Acta Zool.

[268] Barthlott W, Neinhuis C (1997). Purity of the sacred lotus, or escape from contamination in biological surfaces. Planta.

[269] Helbig R, Nickerl J, Neinhuis C, Werner C (2011). Smart skin patterns protect springtails. PLoS One.

[270] Hensel R, Finn A, Helbig R (2013). Biologically inspired omniphobic surfaces by reverse imprint lithography. Adv Mater.

[271] Hensel R, Helbig R, Aland S (2013). Wetting resistance at its topographical limit: the benefit of mushroom and serif T structures. Langmuir.

[272] Epstein AK, Pokroy B, Seminara A, Aizenberg J (2011). Bacterial biofilm shows persistent resistance to liquid wetting and gas penetration. Proc Natl Acad Sci U S A.

[273] Bohn HF, Federle W (2004). Insect aquaplaning: Nepenthes pitcher plants capture prey with the peristome, a fully wettable water-lubricated anisotropic surface. Proc Natl Acad Sci.

[274] Kelleher SM, Habimana O, Lawler J, et al (2015) Cicada wing surface topography: an investigation into the bactericidal properties of nanostructural features. ACS Appl Mater Interfaces acsami.5b08309. 10.1021/acsami.5b08309 10.1021/acsami.5b08309 26551558

[275] Diu T, Faruqui N, Sjöström T (2014). Cicada-inspired cell-instructive nanopatterned arrays. Sci Rep.

[276] Ivanova EP, Hasan J, Webb HK (2013). Bactericidal activity of black silicon. Nat Commun.

[277] Li X (2015). Bactericidal mechanism of nanopatterned surfaces. Phys Chem Chem Phys.

[278] Bandara CD, Singh S, Afara IO (2017). Bactericidal effects of natural nanotopography of dragonfly wing on Escherichia coli. ACS Appl Mater Interfaces.

[279] Hasan J, Raj S, Yadav L, Chatterjee K (2015). Engineering a nanostructured “super surface” with superhydrophobic and superkilling properties. RSC Adv.

[280] Fisher LE, Yang Y, Yuen M-F (2016). Bactericidal activity of biomimetic diamond nanocone surfaces. Biointerphases.

[281] Feng G, Cheng Y, Wang SY (2015). Bacterial attachment and biofilm formation on surfaces are reduced by small-diameter nanoscale pores: how small is small enough?. npj Biofilms Microbiomes.

[282] Feng G, Cheng Y, Wang SY (2014). Alumina surfaces with nanoscale topography reduce attachment and biofilm formation by Escherichia coli and Listeria spp. Biofouling.

[283] Bierbaum S, Mulansky S, Bognár E (2018). Osteogenic nanostructured titanium surfaces with antibacterial properties under conditions that mimic the dynamic situation in the oral cavity. Biomater Sci.

[284] Ionescu AC, Brambilla E, Azzola F (2018). Laser microtextured titanium implant surfaces reduce in vitro and in situ oral biofilm formation. PLoS One.

[285] Miao X, Wang D, Xu L (2017). The response of human osteoblasts, epithelial cells, fibroblasts, macrophages and oral bacteria to nanostructured titanium surfaces: a systematic study. Int J Nanomed.

[286] Ferraris S, Cochis A, Cazzola M (2019). Cytocompatible and anti-bacterial adhesion nanotextured titanium oxide layer on titanium surfaces for dental and orthopedic implants. Front Bioeng Biotechnol.

[287] Moyano DF, Saha K, Prakash G (2014). Fabrication of corona-free nanoparticles with tunable hydrophobicity. ACS Nano.

[288] Krause JE, Brault ND, Li Y (2011). Photoiniferter-mediated polymerization of zwitterionic carboxybetaine monomers for low-fouling and functionalizable surface coatings. Macromolecules.

[289] Carr LR, Zhou Y, Krause JE (2011). Uniform zwitterionic polymer hydrogels with a nonfouling and functionalizable crosslinker using photopolymerization. Biomaterials.

[290] Chen S, Liu L, Jiang S (2006). Strong resistance of oligo(phosphorylcholine) self-assembled monolayers to protein adsorption. Langmuir.

[291] Lewis AL (2000). Phosphorylcholine-based polymers and their use in the prevention of biofouling. Colloids Surfaces B Biointerfaces.

[292] Youngblood JP, Andruzzi L, Ober CK (2003). Coatings based on side-chain ether-linked poly(ethylene glycol) and fluorocarbon polymers for the control of marine biofouling. Biofouling.

[293] Kirillova A, Marschelke C, Friedrichs J (2016). Hybrid hairy Janus particles as building blocks for antibiofouling surfaces. ACS Appl Mater Interfaces.

[294] Aggarwal N, Lawson K, Kershaw M (2009). Protein adsorption on heterogeneous surfaces. Appl Phys Lett.

[295] Takahara A, Hara Y, Kojio K, Kajiyama T (2002). Plasma protein adsorption behavior onto the surface of phase-separated organosilane monolayers on the basis of scanning force microscopy. Colloids Surfaces.

[296] Fang JY, Knobler CM (1996). Phase-separated two-component self-assembled organosilane monolayers and their use in selective adsorption of a protein. Langmuir.

[297] Wong SY, Han L, Timachova K (2012). Drastically lowered protein adsorption on microbicidal hydrophobic/hydrophilic polyelectrolyte multilayers. Biomacromolecules.

[298] Jackson AM, Myerson JW, Stellacci F (2004). Spontaneous assembly of subnanometre-ordered domains in the ligand shell of monolayer-protected nanoparticles. Nat Mater.

[299] Baxamusa SH, Gleason KK (2009). Random copolymer films with molecular-scale compositional heterogeneities that interfere with protein adsorption. Adv Funct Mater.

[300] Hung A, Mwenifumbo S, Mager M (2011). Ordering surfaces on the nanoscale: implications for protein adsorption. J Am Chem Soc.

[301] Paterlini TT, Nogueira LFB, Tovani CB (2017). The role played by modified bioinspired surfaces in interfacial properties of biomaterials. Biophys Rev.

[302] Naha PC, Liu Y, Hwang G, et al (2019) Dextran coated iron oxide nanoparticles as biomimetic catalysts for localized and pH-activated biofilm disruption. ACS Nano acsnano.8b08702. 10.1021/acsnano.8b08702 10.1021/acsnano.8b08702 PMC7059368 30642159

